# Microstructure, Oxidation Resistance and High-Temperature Tribological Properties of CoCrWReAl-xRu-TiC Eutectic Alloys

**DOI:** 10.3390/ma19214684

**Published:** 2026-11-03

**Authors:** Alina Yurchuk, Oleksandr Tisov, Myroslav Kindrachuk, Mykhaylo Pashechko, Tetiana Cherepova

**Affiliations:** 1 Air Transport Management Department, Faculty of Transport, Management and Logistics, National University “Kyiv Aviation Institute”, 03058 Kyiv, Ukraine; 2 School of Aerospace Engineering, Xi’an Jiaotong University, Xi’an 710049, China; 3 Department of Applied Mechanics and Materials Engineering, National University “Kyiv Aviation Institute”, 03058 Kyiv, Ukraine; 4 Department of Technical Computer Science, Faculty of Mathematics and Information Technology, Lublin University of Technology, 20-618 Lublin, Poland; 5 G.V. Kurdiumov Institute for Metal Physics, National Academy of Science of Ukraine, 03142 Kyiv, Ukraine

**Keywords:** REM, high-temperature wear resistance, oxidation resistance, Ru alloying, cobalt-based eutectic alloys

## Abstract

CoCrWReAl-xRu-TiC eutectic alloys (x = 0, 1, 2.5, and 5 wt.%) were fabricated in a tungsten arc furnace under a protective argon atmosphere using pure metals and amorphous graphite. We examined the phase composition and microstructure of the samples and triboinduced layers using SEM-EDS and XRD. DSC was used to determine their melting temperature. The oxidation resistance tests were performed using a thermogravimetric analyzer under isothermal conditions at 1100 °C for 100 h. Oxidation damage was estimated from weight-gain graphs, oxide layer thickness, the relative area of internally oxidized material, and exterior visual analysis. Wear resistance was tested at 1100 °C under dry ball-on-disc sliding conditions for 2 h against a 10 mm Si_3_N_4_ ceramic ball at a normal load of 100 N. Increasing Ru content initially refines the carbide structure and makes their distribution in the cobalt matrix more uniform, increasing alloy hardness and wear resistance. At higher concentrations, carbide morphology changes from fine particulate to coarse split-dendritic, increasing their microhardness and intensifying abrasive wear. The oxidation resistance decreases with increasing Ru weight fraction. The 1 wt.% Ru alloy provided the most favorable balance of melting temperature, oxidation resistance, mechanical, and tribological properties.

## 1. Introduction

Cobalt-based alloys are widely used as tribological coatings for turbine blades [1,2], stator vanes [3,4], and connecting joints in aircraft air-conditioning and engine start systems [5,6]. They exhibit excellent strength and wear resistance at elevated temperatures and have a long history of successful application. For decades, these alloys have been used in some of the most advanced aircraft ever manufactured. Despite their successful implementation, important research gaps remain, particularly regarding the effects of rare-earth and other expensive alloying elements. Historically, the high cost of these elements limited their use, and available experimental equipment often lacked the capability to characterize their influence on the tribological performance of coatings and bulk materials.

Several studies have investigated the Co-Cr-TiC system, but carbide morphology, size, and distribution have received comparatively little attention, except in a few publications [7,8,9]. Moreover, these studies do not focus specifically on the role of carbides, how alloying elements affect their size and distribution, or the alloy’s overall oxidation resistance. Most studies focus on solid-solution [10,11,12] and precipitation-strengthened alloys [13,14] and pay little attention to carbide strengthening. Carbides improve wear resistance but reduce oxidation resistance and require additional solid-solution strengthening to retain the carbides.

Data on the effects of rare-earth elements on the tribological properties of Co-TiC alloys remain limited, largely due to the difficulties associated with their fabrication. In [15], the authors modified Tribaloy 800 with 4 wt.% Re and investigated its oxidation resistance at 800 °C and 1000 °C. Adding Re led to a thicker oxidation scale and deeper inter-dendritic oxidation, with increased weight gain at 800 °C but decreased weight gain at 1000 °C. In addition, 4 wt.% Re promoted oxide spallation. However, they did not examine the effect of Re on tribological properties. Moreau et al. [16] studied the influence of adding up to 15 wt.% Re on the properties of Co-Cr-Ta-Ti-C alloys. They found that increasing the Re content above 10 wt.% raised the melting temperature but significantly reduced the oxidation resistance. Smolina and Kibiela [17] investigated laser surface alloying of Stellite 6 with Re, achieving 11–17 wt.% Re in the surface layer. This treatment substantially improved the hardness and wear resistance of Stellite 6. Li et al. [18] also examined the effect of Re on novel cobalt superalloys and showed that 1, 3, and 5 at.% Re promoted precipitation of a second phase from the cobalt solid solution, thereby increasing strength. Yang et al. [19] reported that 2 wt.% Re in Co-Ni superalloys increased lattice mismatch and dislocation density, redistributed elements within the alloy, and reduced intergranular cracking, although some thermal properties deteriorated. Overall, although Re addition may decrease oxidation resistance, it significantly improves mechanical properties [20,21] and remains a promising route for developing cobalt superalloys.

Recently, ruthenium has attracted considerable attention as a promising alloying element for superalloys [22,23]. Owing to its similarity to rhenium, Ru is frequently used as an alloying addition in nickel-based superalloys. It increases the volume fraction and modifies the gamma-prime phase morphology [24], thereby enhancing wear resistance. These effects suggest that Ru may also influence elemental redistribution in solid solution and promote carbide precipitation. Another study reported substantial strengthening of solid-solution alloys with 0.6% Ru, accompanied by a 31–83% increase in wear resistance [25]. However, these tests were conducted at room temperature, and they did not investigate high-temperature wear behavior. In addition, Ru reduces Re partitioning, thereby decreasing the propensity for brittle phase formation [26].

Hango et al. studied the 0.3 and 0.6 wt.% of rhenium on the properties of ULTIMET^TM^ alloys [27]. Minor additions of REM (rare-earth metals) noticeably improve their tribological properties. Sauza et al. [28] studied Co-9.4Al-7.5W-2.1Ru at.%. A 2 at.% Ru addition to a Co-Al-W superalloy was shown to preferentially partition to the γ′ phase without significantly affecting its volume fraction or coarsening kinetics. However, prolonged aging at 900 °C led to a rapid, discontinuous transformation of the γ/γ′ microstructure into a lamellar mixture of Co_3_W, γ, and B2 phases, indicating that Ru can substantially influence the long-term phase stability of Co-based superalloys.

Most published studies on superalloys have focused primarily on mechanical strength, creep resistance, and oxidation behavior, examining the effects of Ru or Re on high-temperature performance. These investigations have also commonly included microhardness measurements and microstructural characterization. However, the high-temperature wear behavior of high-Re alloys containing Ru remains underexplored, and the influence of Ru on the microstructure–property relationships of these materials has not been systematically established. The present study addresses this gap by investigating the effect of Ru additions on CoCrWReAl-xRu-TiC alloys, where x = 0, 1, 2.5, and 5. Particular emphasis is placed on correlating the Ru content with the alloy microstructure, carbide morphology and distribution, phase constitution of both the alloy and triboinduced surface layers, oxidation behavior, microhardness, bulk hardness, and most importantly, high-temperature tribological performance. This integrated approach provides new insight into the role of Ru in governing the wear and oxidation response of high-Re Co-based alloys reinforced with TiC.

## 2. Materials and Methods

**Figure 1:**
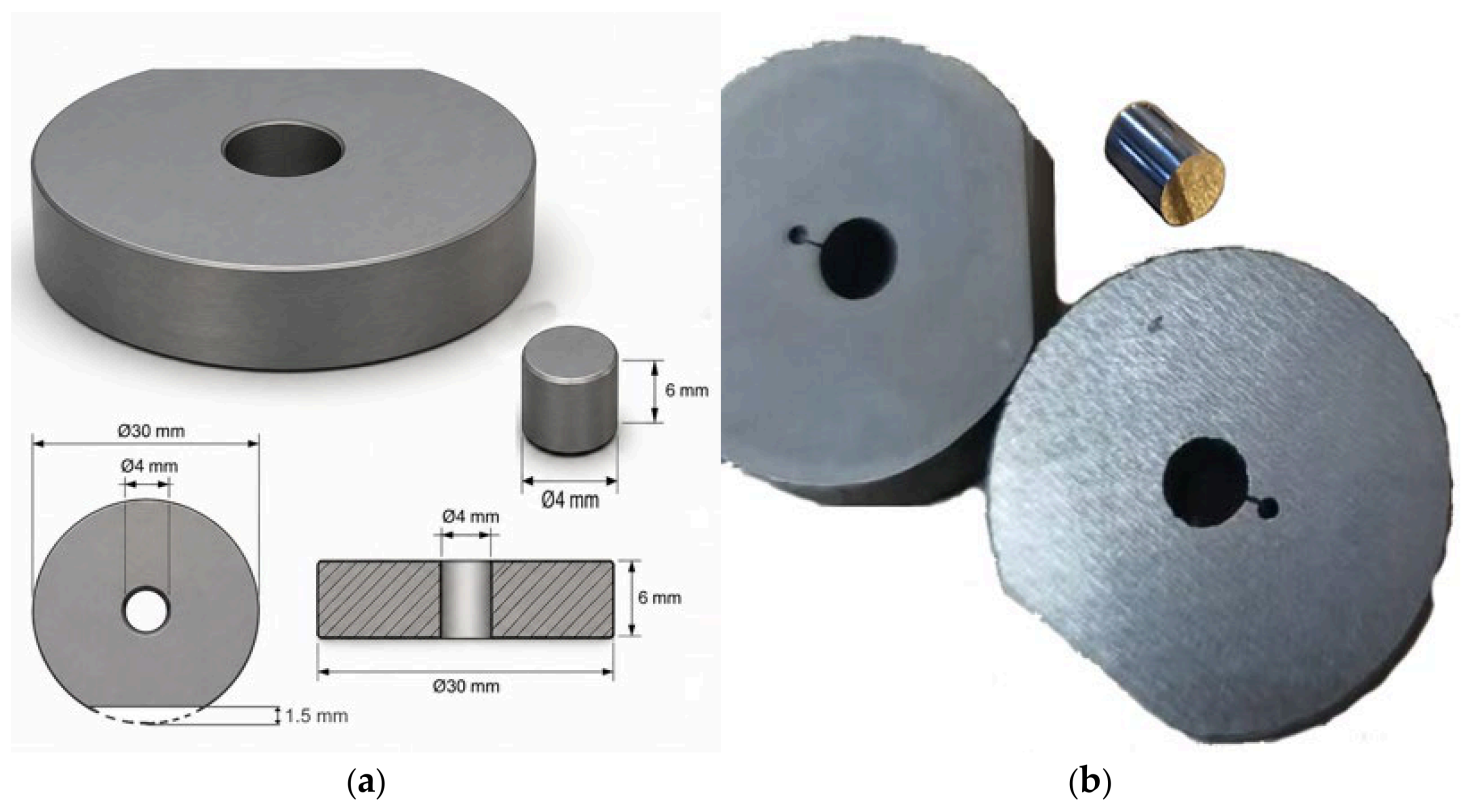
Drawing of the samples (**a**) and fabricated samples ready for the tests (**b**).

For alloy casting, we used 99.9% pure Co, Cr, W, Re, Al, and Ti granules (3–5 mm particle size), Ru grains (1–3 mm), and 200-mesh amorphous graphite powder (Beijing Institute of Advanced Materials, Beijing, China). The alloying components were weighed with an accuracy of 0.01 g to produce ingots with a total mass of 200–220 g, 3 ingots per composition. To prevent dispersion during arc ignition and to ensure a more accurate alloy composition, the graphite powder was wrapped in aluminum foil. Melting was carried out in a protective argon atmosphere at a pressure of 10^−5^ Pa using a self-assembled arc-melting furnace equipped with a tungsten electrode (MZ-1250, Beijing Yijin New Materials Technology Co., Ltd., Beijing, China). Each alloy was remelted five times in succession to promote compositional homogeneity. For tribological testing, we sectioned each ingot into 30 × 6 mm discs using a wire-EDM machine (DK7750, Suzhou Hanqi Equipment Co., Suzhou, China) in dielectric fluid. Only the discs from the middle of the ingot were used for further tests, as this part had the most uniform elemental distribution and was less oxidized—3 discs per composition. We also used microdrilling and wire EDM (Electro-Discharge Machining) to prepare 4-mm diameter pins for thermogravimetric testing, 3 samples per ingot, 9 samples per composition. The sample geometry and pin are shown in Figure 1. Table 1 presents the elemental contents of the alloying elements in the fabricated samples.

Metallographic specimens and cross-sections of the alloys after oxidation resistance testing were prepared for scanning electron microscopy (SEM) analysis using a GP2000A automatic metallographic polishing machine (Trojan, Suzhou, China), followed by sequential grinding with SiC abrasive papers and polishing with diamond suspensions and colloidal silica.

The surface morphology, microstructure, oxidation behavior, and internal oxidation characteristics of the materials were examined using a Gemini Sigma 300 (Carl Zeiss Microscopy GmbH, Oberkochen, Germany) and a SU3500 (Hitachi High-Tech Corporation, Tokyo, Japan) scanning electron microscope, both equipped with an energy-dispersive X-ray spectroscopy (EDS) system (Oxford Instruments, Abingdon, UK). We performed oxidation resistance tests using an HTG-3 thermogravimetric analyzer (TGA) for 100 h at 1100 °C in isothermal conditions. The melting temperatures of the alloys were determined by differential scanning calorimetry (DSC) using an HSC-5 instrument based on 3 measurements for each alloy. Beijing Hengjiu Equipment, Beijing, China, manufactured both thermal analysis devices.

Vickers hardness measurements were performed using a Vickers hardness tester (XHV-1000T-CCD, Suzhou Nanguang Electronic Technology Co., Ltd., Suzhou, China) with a load of 10 g, or 0.098 N and a dwell time of 10 s (HV_0.01_), with 30 measurements per phase (carbides and solid solution SS) for each alloy. We also used its built-in optical acquisition system to obtain light microscopy images for an initial assessment of the as-cast microstructure, carbide number, size, and morphology. The hardness was measured with an HRS-150m Rockwell universal hardness tester (Suzhou Nanguang Electronic Technology Co., Ltd., Suzhou, China), conducting 10 equally spaced measurements along each disc diameter and 30 measurements per alloy.

To determine carbide morphology, size, and quantity for each composition, we acquired ×100 light micrographs from the center of three disc samples. We then performed additional imaging by taking three equidistant photographs along the 3, 6, 9, and 12 o’clock directions, resulting in 13 images per disc (39 images per alloy). Each image had a field of view of 0.7 × 0.52 mm, corresponding to an area of 0.364 mm^2^, resulting in a total analyzed surface area of 14.2 mm^2^ per alloy. We analyzed carbide grain quantity and size, and the area of Al-rich oxidation beneath the external scale, using the open-source software Fiji (version 2.16.0/1.54f, National Institutes of Health, Bethesda, MA, USA).

The samples used for friction testing were mirror-polished using the same procedure as for SEM specimen preparation, 3 samples per alloy. Commercially available high-precision Si_3_N_4_ balls (Jiangsu Zhibang Precision Technology Co., Ltd., Changzhou, Jiangsu, China), with a manufacturer-specified roughness of Ra 0.02 μm and a Vickers hardness of 15.5 GPa, were used as counterbodies. We carried out tribological tests on an MFT5000 tribometer in a pin-on-disc configuration (RTEC Instruments, San Jose, CA, USA) at 1100 °C for 2 h (7200 s). The ball was positioned at a wear track radius of 11 mm and rotated at 210 rpm, corresponding to a sliding distance of 1742 m and a linear velocity of 0.242 m/s. A constant normal load of 100 N was applied throughout the test. We acquired the three-dimensional surface topography images of the wear tracks using a UP3000 optical confocal profilometer (RTEC Instruments, San Jose, USA) and determined the corresponding wear volumes using MountainsMap^®^ software (version 10; Digital Surf, Besançon, France).

X-ray diffraction (XRD) spectra were acquired at room temperature from mirror-polished discs (30 × 6 mm) using an X-ray diffractometer (D8 Advance, Bruker, Karlsruhe, Germany), with 3 samples per composition. XRD analysis was performed using Cu Kα radiation with a wavelength of λ = 1.5418 Å (0.15418 nm). Diffraction patterns were recorded over the 2θ range of 35–95° for the polished samples and 30–80° for the friction surfaces. The measurements were conducted with a step size of 0.0112715° and an acquisition time of 0.056 s per step. We qualitatively analyzed the diffraction patterns using the PDF-2 database (ICDD, release 2023, Newtown Square, PA, USA).

## 3. Results and Discussion

### 3.1. Phase Analyses of the Alloys Before the Friction Test

**Figure 2:**
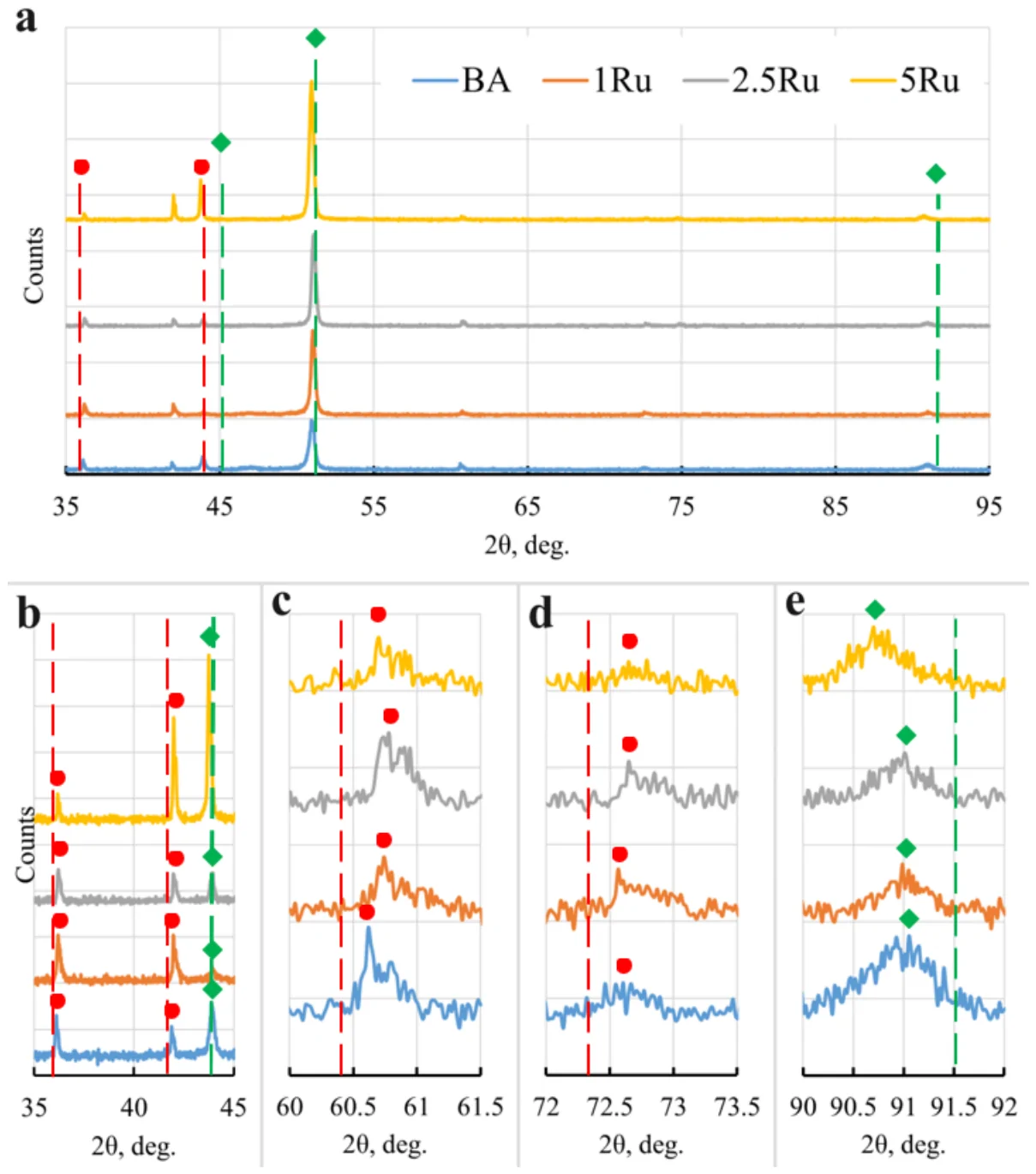
XRD patterns of the polished samples’ surfaces before the tests: (**a**) full spectra; (**b**–**e**) enlarged sections of the samples’ spectra. 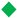 Co-reach FCC, 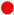 TiC.

Figure 2a shows the XRD patterns of the polished sample surfaces, including the full spectra, and indicates two major phases: cobalt-based solid solution Co_FCC_ (PDF 01-086-9011) and titanium carbide TiC (PDF 00-031-1400). Table 2 and Table 3 provide the complete list of reflections identified for the investigated alloys.

Qualitative analyses of the XRD patterns [29,30] revealed that all as-cast alloys contained cobalt-based FCC solid solutions and titanium carbides. The discernible reflections fall into two groups: those corresponding to titanium carbide and those associated with the face-centered cubic (FCC) cobalt-based solid solution containing the alloying elements. No additional peaks indicative of the formation of new phases were detected. Table 2 and Table 3 present the complete list of observed peaks and their assignments to the corresponding phases. This does not necessarily imply that such phases are entirely absent; however, if present, their volume fraction is negligibly small and may therefore be disregarded. Accordingly, ruthenium most likely dissolves in the cobalt matrix without forming undesirable intermetallic phases.

For titanium carbide (Table 2), only minor variations in the 2θ positions were observed. These variations may indicate changes in TiC composition caused by W and Re diffusion into its crystal lattice, suggesting the formation of a complex carbide of the type (Ti, W, Re)C. The Co_FCC_ peaks also exhibited shifts, as shown in Table 3. The decrease in diffraction angles at high Ru contents corresponds to increased interplanar spacing and expansion of the Co_FCC_ lattice. Thus, Ru stabilizes and modifies the Co_FCC_ matrix without generating additional undesirable phases.

Overall, the cobalt-based solid solution was the predominant phase across all investigated alloys, regardless of Ru content. Its principal reflections correspond to the (111), (200), and (311) crystallographic planes, with the (111) reflection remaining the most intense for all specimens.

Variation in Ru content affects the intensities of these reflections, most pronounced in the alloy containing 5 wt.% Ru. In addition, as shown in Figure 2 and Table 2 and Table 3, the Co_FCC_ reflections shifted towards lower 2θ angles, particularly the (111) and (311) reflections. According to Bragg’s law, this shift indicates increased interplanar spacing and, consequently, a larger lattice parameter in the FCC phase. Therefore, Ru dissolution in the cobalt crystal lattice slightly modifies its structure but does not form rhenium-rich phases.

### 3.2. The Effect of Ruthenium on the Melting Temperature

**Figure 3:**
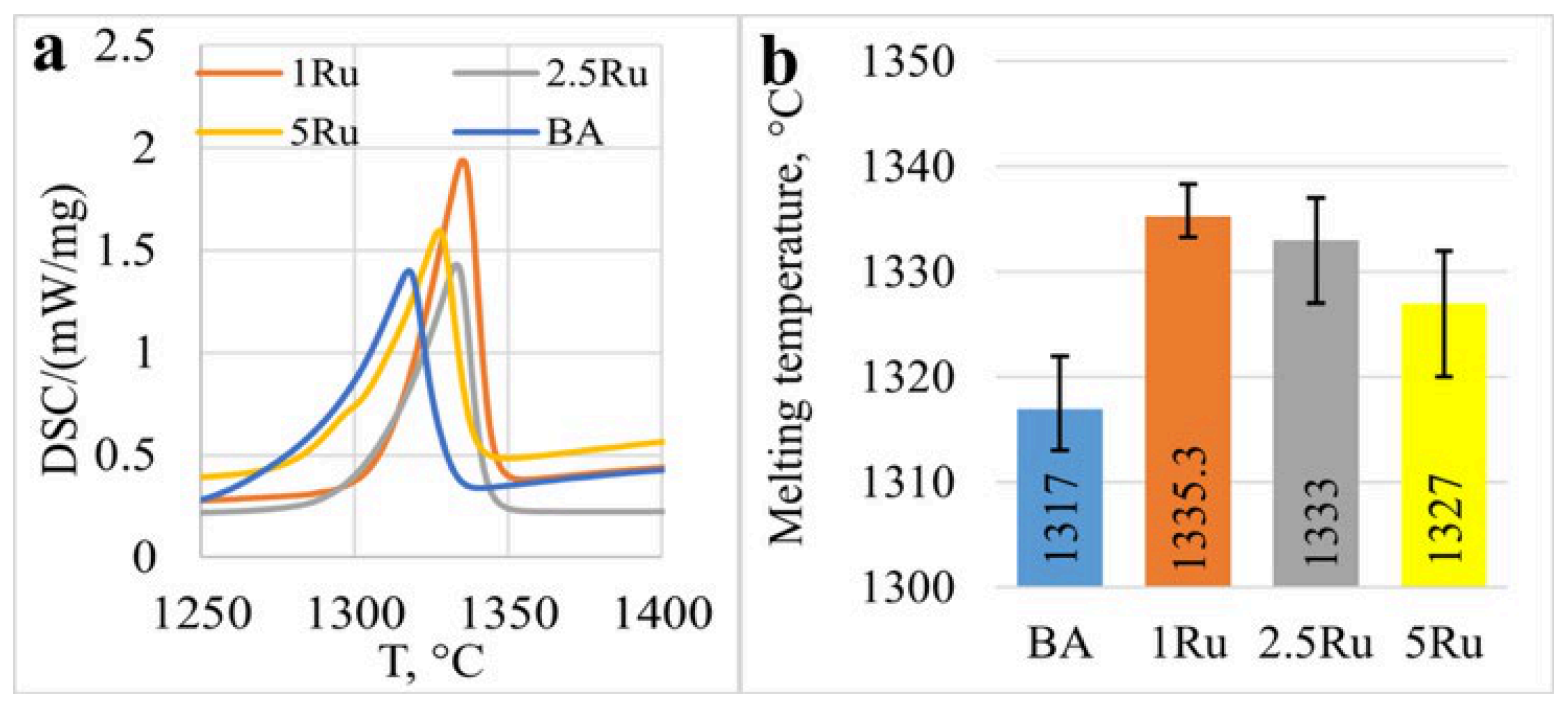
DSC curves (**a**) and histogram of the melting points of the studied alloys (**b**).

Figure 3 presents the DSC curves and the melting-temperature histogram of the investigated alloys. Based on three measurements, the melting temperature of the base alloy, which was not modified with ruthenium, was 1317 °C. The addition of 1 wt.% Ru increased the melting temperature by 18.3 °C, representing the largest change among the investigated alloys, to 1335.3 °C. The addition of 2.5 wt.% Ru decreased the melting temperature to 1333 °C, which was 2.3 °C lower than that of the alloy containing 1 wt.% Ru, but still 16 °C higher than that of the base alloy. Increasing the Ru content to 5 wt.% further reduced the melting temperature to 1327 °C, which remained 10 °C higher than that of the base alloy. Thus, the effect of Ru is non-monotonic: the maximum melting temperature was achieved at 1 wt.% Ru, after which it gradually decreased with increasing Ru concentration (Figure 3a,b). Therefore, the addition of 1 wt.% Ru has a relatively limited effect on the crystal structure of cobalt while increasing its melting temperature [31].

Ruthenium has a high melting temperature and is highly soluble in cobalt. At low concentrations, it strengthens interatomic bonds and stabilizes the cobalt matrix without the formation of new phases. This increases the melting temperature. At the same time, such a small amount of Ru is unlikely to substantially affect diffusion processes or the overall function of the other alloying elements. At higher Ru concentrations, its interactions with Cr, W, Re, and Al become more pronounced. Further increases in Ru content in the solid solution lead to greater compositional inhomogeneity, liquation, and new phase formation. Consequently, coarser carbides are formed. This creates microregions with a lower melting temperature, thereby lowering the alloy’s overall melting temperature.

Consequently, the melting temperatures of the alloys containing 2.5 and 5 wt.% Ru were lower than those of the alloy containing 1 wt.% Ru.

### 3.3. Microstructure Examinations

**Figure 4:**
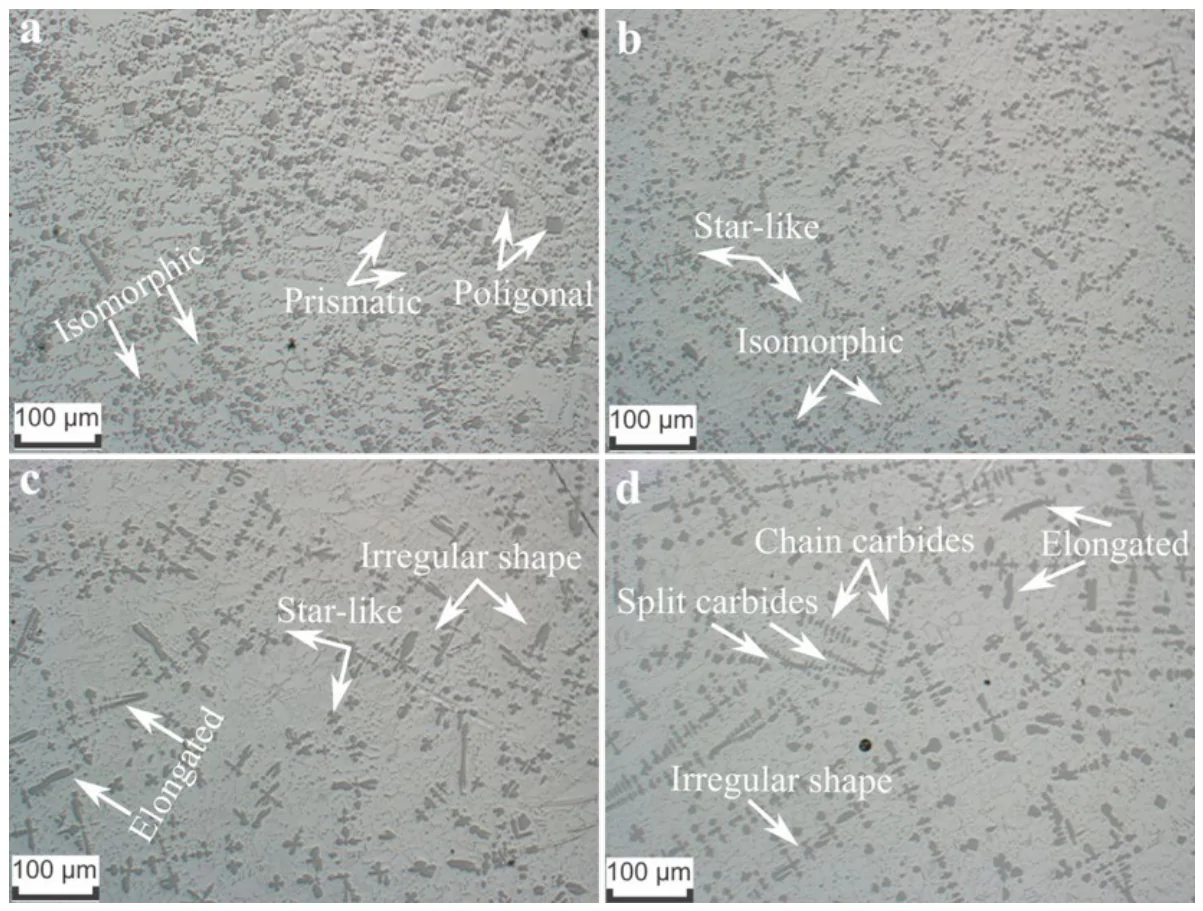
Light microscopy acquired microphotographs of the base alloy BA (**a**) and alloys containing 1, 2.5, and 5 wt.% of Ru (**b**), (**c**), and (**d**), respectively.

Figure 4 presents optical micrographs of the microstructures of the investigated alloys. Particular attention should be paid to the size and distribution of the titanium carbide particles over the observed surface, which are non-uniform and clearly depend on Ru content in both particle size and morphology. For this assessment, low-magnification micrographs (×100 in the present case) are preferable because they provide a larger field of view, although some of the smallest particles may not be resolved. Table 4 provides a comparative characterization of the carbide particles, together with their calculated number density per 1 mm^2^ of the alloy surface.

All alloys in Figure 4 showed a bright matrix phase and darker carbide particles. Based on the alloy compositions (Table 1), previous studies [32,33,34,35], and X-ray microanalysis results, the bright phase is a solid solution of the alloying elements in the cobalt matrix, whereas the dark phase corresponds to alloyed titanium carbides. The base alloy (Figure 4a) contains the highest number of fine carbide particles, distributed between larger carbides and located both along the cobalt grain boundaries and within the grains. This distribution may indicate their formation through a precipitation reaction, i.e., the precipitation of carbides from a previously solidified supersaturated solid solution. Most carbides exhibited a rounded, isometric morphology. We also observed prismatic and polygonal carbides. The isometric carbides ranged from 5 to 9 μm, whereas polygonal particles could reach 30–40 μm (Figure 4a and Table 4).

The addition of 1 wt.% Ru substantially modified the carbide morphology, whereas the matrix phase remained essentially unchanged. Visually, this alloy exhibited the finest carbide population, with predominantly isometric carbides measuring 4–6 μm (Figure 4b, Table 4), accompanied by individual irregularly shaped particles reaching 20–30 μm. At the same time, the number of elongated carbides decreased. This may indicate that Ru addition suppresses carbide growth and/or promotes carbide nucleation during solidification. Both mechanisms would refine carbides, increase their number density per 1 mm^2^ (Table 4), and reduce the fraction of fine particles precipitated from the solid state.

At 2.5 wt.% Ru, the carbide morphology underwent further modification. The characteristic carbide size increased to 6–10 μm (Figure 4c, Table 4), while individual elongated carbides could reach 40–60 μm. Both the number and size of star-shaped and irregularly shaped carbides increased. Small carbide clusters, which were not apparent in the BA and 1Ru specimens, also formed, along with carbide coalescence that produces highly elongated particles (Figure 4c).

In the alloy containing 5 wt.% Ru, the tendency toward carbide coarsening and coalescence became even more pronounced. Simultaneously, the fraction of fine carbides decreased, while the proportion of coarse particles increased, and their calculated number density decreased to approximately 700–1000 particles/mm^2^ (Figure 4d, Table 4). The average carbide size increased to 8–13 μm, while some particles reached 50–70 μm, representing the largest carbide dimensions among the studied alloys. The spatial distribution of carbides within the matrix became increasingly heterogeneous. Carbide chains and split carbides were observed, suggesting a hypereutectic-type solidification structure, whereas the BA, 1Ru, and 2.5Ru alloys exhibited predominantly eutectic and hypoeutectic structures (Figure 4a–c, Table 4).

**Figure 5:**
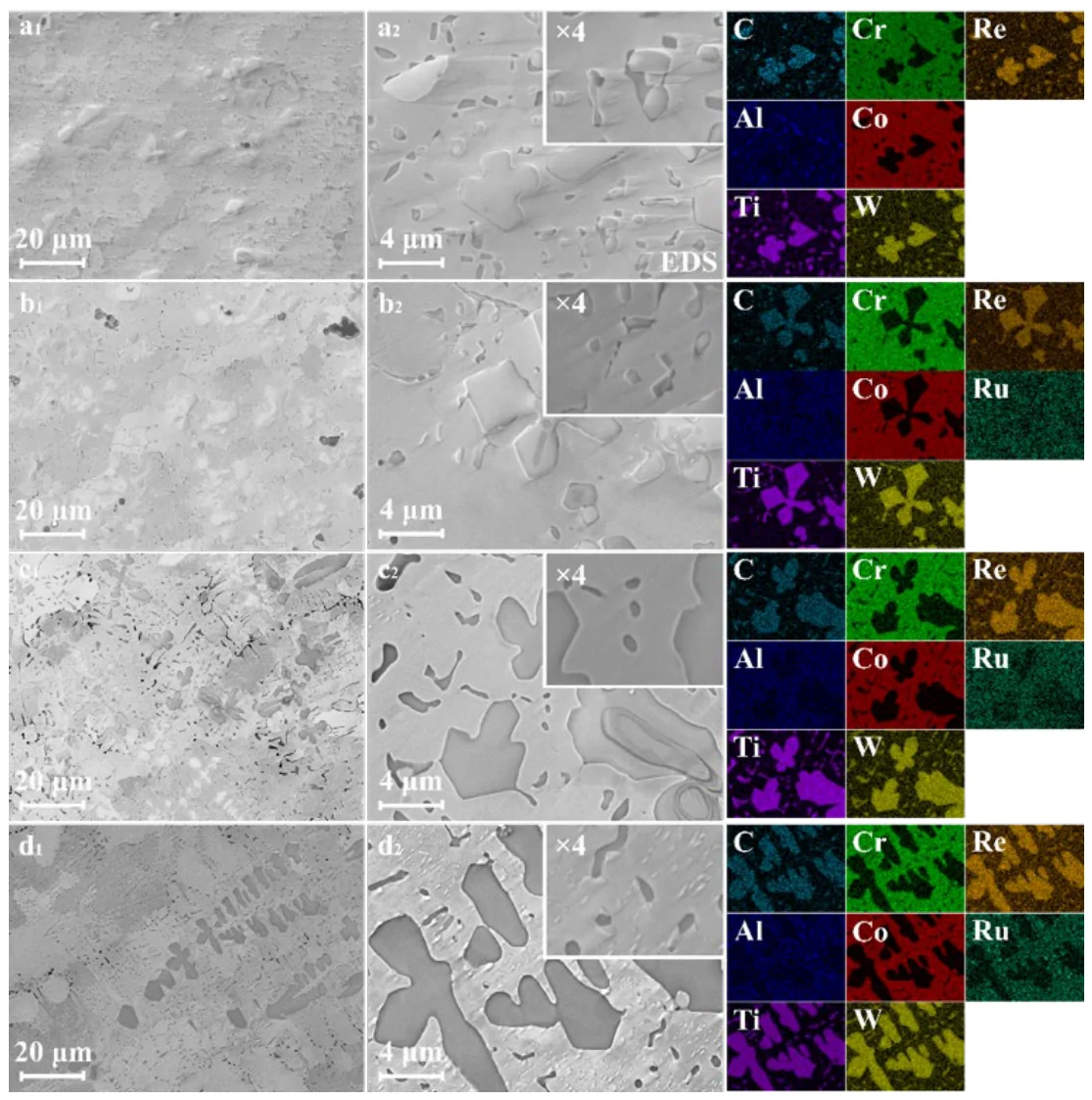
Scanning electron microscopy micrographs of the basic alloy BA (**a_1_**,**a_2_**), and alloys containing 1, 2.5, and 5 wt.% Ru (**b_1_**,**b_2_**), (**c_1_**,**c_2_**), and (**d_1_**,**d_2_**), respectively, with corresponding EDS elemental mappings.

Figure 5 shows the SEM microstructures of the studied alloys with corresponding EDS elemental maps, and Table 5 presents the measured surface elemental composition (wt.%). The inserts in Figure 5(a_2_–d_2_) show the morphology of fine TiC crystals, which are between the coarser ones. These carbides were not visible in Figure 4 because the magnification was insufficient. Notably, the alloy containing 1 wt.% Ru exhibited the highest number of these carbides, the smallest carbide size, and the most uniform distribution. This is expected to positively affect the material’s mechanical and tribological properties. Overall, the carbide grain morphology and size are consistent with Figure 4 and Table 4.

The alloy microstructure consists of two main phases: a cobalt-based solid solution containing the alloying elements and carbide particles (Figure 5, EDS mapping). In all the investigated alloys, tungsten diffusion into the carbide phase was detected, whereas rhenium diffusion was less pronounced. At the same time, the particle morphology remained typical of titanium carbides. Therefore, it is more accurate to describe the carbide phase not as stoichiometric TiC, but as a doped, presumably non-stoichiometric titanium carbide with the composition (Ti, W, Re)C.

This result confirms the assumption regarding the causes of the shifts in the Bragg angles observed in the X-ray diffraction patterns of the alloys (Figure 2, Table 2). According to the elemental distribution maps, the tungsten signals are more intense and more clearly localized within the carbide phase. This indicates that tungsten replaces titanium more readily than rhenium does. Consequently, W most likely contributes more to changes in the carbide lattice parameters than Re.

In other regions of the alloys, W and Re are distributed relatively uniformly, with no localized areas of increased concentration. This suggests that under the conditions investigated, these elements predominantly dissolve in the cobalt matrix and do not form separate intermetallic phases. This interpretation is consistent with the X-ray diffraction results, which revealed no additional phases.

The cobalt and aluminum were also uniformly distributed throughout the base alloy matrix. These elements may form intermetallic compounds, including Co_3_Al. According to the equilibrium phase diagram of the cobalt-aluminum system, Co_3_Al forms at an Al concentration of 6% or higher. High chromium concentration can shift the equilibrium toward lower aluminum concentrations. However, in the present case (Table 1 and Table 5), the aluminum concentration was insufficient to form this phase. Figure 5 provides no evidence of localized, coincident increases in cobalt and aluminum concentrations. Therefore, these intermetallic compounds did not form in the studied alloys.

The measured concentrations of the alloying elements (Table 5) generally matched the nominal values (Table 1), with deviations typical of local EDS analysis. Analysis of the chromium and aluminum distribution maps also revealed no regions enriched in both elements simultaneously. This indicates that oxide inclusions are absent, which might otherwise form if the starting materials were oxidized or if the melting procedure had not been properly controlled, since aluminum acts as a deoxidizer in many metallurgical processes. Thus, chromium-containing intermetallic compounds were not formed either.

The largest discrepancy was observed for Re: its measured content was only 2.2–2.6 wt.%, whereas the nominal concentration in all alloys was 6 wt.%. Its content was well below the nominal 6%. Meanwhile, the W content was much higher than nominal. This is a common error reported by Zibrov et al. [36] due to energy-spectrum overlap and thus misindexing of W and Re when both are present in alloys simultaneously. The characteristic X-ray lines of W and Re are closely spaced and partially overlap, which can lead to significant uncertainty and systematic redistribution of spectral intensity between these elements during conventional EDS peak deconvolution [37]. Typically, measured W values are much higher than actual values, and measured Re values are lower [38]. Therefore, the W and Re concentrations obtained in the present study by SEM-EDS should be considered semi-quantitative rather than absolute, particularly for local measurements.

The measured chromium and aluminum concentrations are likewise lower than their nominal values. In the BA and 5Ru alloys, the titanium content exceeded the nominal value by 1.53 and 1.29 wt.%, respectively, whereas in the other two alloys, it was lower by 0.39 and 0.17 wt.%, respectively. Because Re and W partially substitute Ti in the carbides, this may also explain the lower measured W and Re content in the alloys. The measured increase in C content may be due to the typically low EDS accuracy for light elements.

The most important aspect of this section is investigating the uniformity of ruthenium distribution in the alloys. Examination of the alloy micrographs from top to bottom (Figure 5(a_1_–d_1_)) revealed an increase in the texture and contrast of the grains of the matrix phase with increasing ruthenium content. This effect is attributed to a reduction in the stacking-fault energy (SFE) of the cobalt matrix caused by ruthenium alloying. As a result, the crystallization rate and element redistribution within the alloy grains change [39,40,41,42]. Thus, the darker grains may contain higher concentrations of lighter elements. This assumption is a working hypothesis that we will investigate in future publications.

The distribution of Ru (Figure 5) was uniform: it was present in the alloy matrix as part of the solid-solution phase, together with the other alloying elements. Unlike W and Re, it showed no noticeable incorporation into the carbides.

The base alloy BA contained no ruthenium. The grain boundaries of the solid-solution phase were generally visible, although in the right-hand part of the image (Figure 5(a_1_)), they merged into one continuous field. The addition of 1 wt.% Ru caused only minor changes in the grain structure of the matrix and did not alter the matrix grain size, although it made the grain boundaries more distinct and the grains themselves more contrasting. Interestingly, the ruthenium contrast was similar in both the matrix and the carbides, even though no regions with an increased ruthenium concentration were visible. Since ruthenium generally does not accumulate in carbides, as indicated by the literature, this observation may be attributed to mis-indexing caused by the high tungsten and rhenium contents combined with the low ruthenium concentration.

The addition of 2.5 wt.% Ru increased the contrast of the elemental distribution map, enabling the identification of certain compositional inhomogeneities; however, the overall pattern remained similar to that of the preceding alloy. The ruthenium concentration in the solid solution was clearly substantially higher than in the carbides. This difference was even more pronounced in the alloy containing 5 wt.% Ru. Thus, ruthenium primarily contributes to solid-solution strengthening of the cobalt matrix and exhibits almost no incorporation into the carbides.

In the 2.5Ru and 5Ru alloys, the cobalt grain size also increased considerably, accompanied by enhanced contrast. This behavior may be associated with slower cobalt diffusion at higher ruthenium contents [39,43]. These studies found no new phases in the cobalt- and nickel-based alloys containing up to 10 at.% of Ru: neither intermetallic compounds nor the hexagonal close-packed (HCP) form of Co were formed. This should favorably affect the structural stability and wear resistance of the developed alloys.

To summarize, the addition of 1 wt.% Ru resulted in a pronounced 25–35% refinement of the TiC particles compared with the base alloy (Table 4) while also providing the most uniform carbide distribution. Increasing the Ru content beyond 1 wt.% progressively diminished this refinement effect and promoted significant carbide coarsening, accompanied by a wider particle-size distribution. This effect was particularly pronounced in the alloy containing 5 wt.% Ru, in which the carbides were approximately 30–40% larger than those in the base alloy. Adding up to 5 wt.% ruthenium did not lead to the formation of new phases. Ruthenium predominantly dissolved in the matrix, thereby contributing to solid-solution strengthening.

### 3.4. Durometric Tests

**Figure 6:**
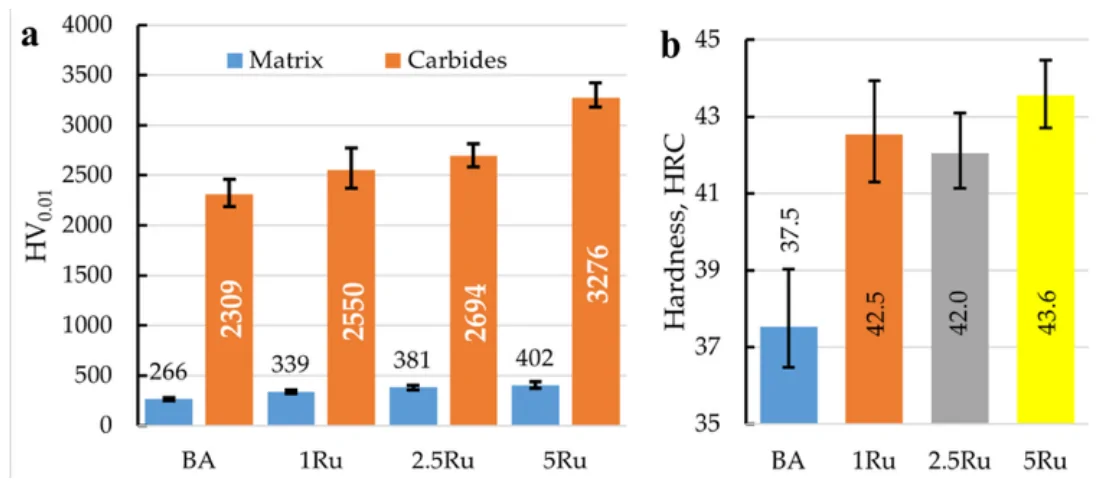
Vickers microhardness (**a**) and HRC hardness (**b**) of the tested samples.

Figure 6a shows the microhardness of the solid-solution phase and carbides in the samples, and Figure 6b shows the bulk material’s Rockwell HRC hardness. Adding Ru increases the microhardness of carbides and the solid solution. Alloying with Ruthenium increased their hardness by 241, 385, and 967 units (10.4, 16.7, and 41.9%) relative to the BA sample.

In absolute values relative to the base alloy, 1 wt.% of Ru increased the matrix microhardness to HV_0.01_ = 339, a 27.4% increase. Further Ru addition yielded a slower increase in microhardness: HV_0.01_ = 381 (43.2%) for 2.5 wt.% of Ru and HV_0.01_ = 402 (51.1%) for 5 wt.% of Ru. Ruthenium addition also changed the carbide morphology and size (see Figure 4 and Figure 5, Table 4), and consequently, the microhardness (Figure 6a). The unstrengthened carbides were HV_0.01_ 2309.

Increased solid-solution strengthening, changes in carbide morphology, and increased microhardness increased the bulk sample hardness; see Figure 6b. This was not as monotonic as for microhardness. We obtained a steep increase for 1Ru, from 37.5 HRC to 42.5 HRC (11.8%); a slight decrease for 2.5Ru to 42 HRC (a 10.7% increase relative to BA); and finally, 5Ru increased the hardness to 43.6 HRC (14%).

Ruthenium changes the diffusion rate within the SS grain, thus might reduce dislocation mobility and produce solid-solution strengthening. Alternating diffusion processes lead to carbide coarsening and thus higher resistance to indenter penetration. This may be true if the carbide distribution is uniform. As shown in Figure 4, as Ru increases, the carbide distribution becomes less ordered, despite increased carbides and solid-solution strengthening; bulk hardness even decreases at 2.5Ru and is slightly higher at 5Ru.

### 3.5. Oxidation Resistance

#### 3.5.1. Oxidation Surface Analyses and Weight Gain

**Figure 7:**
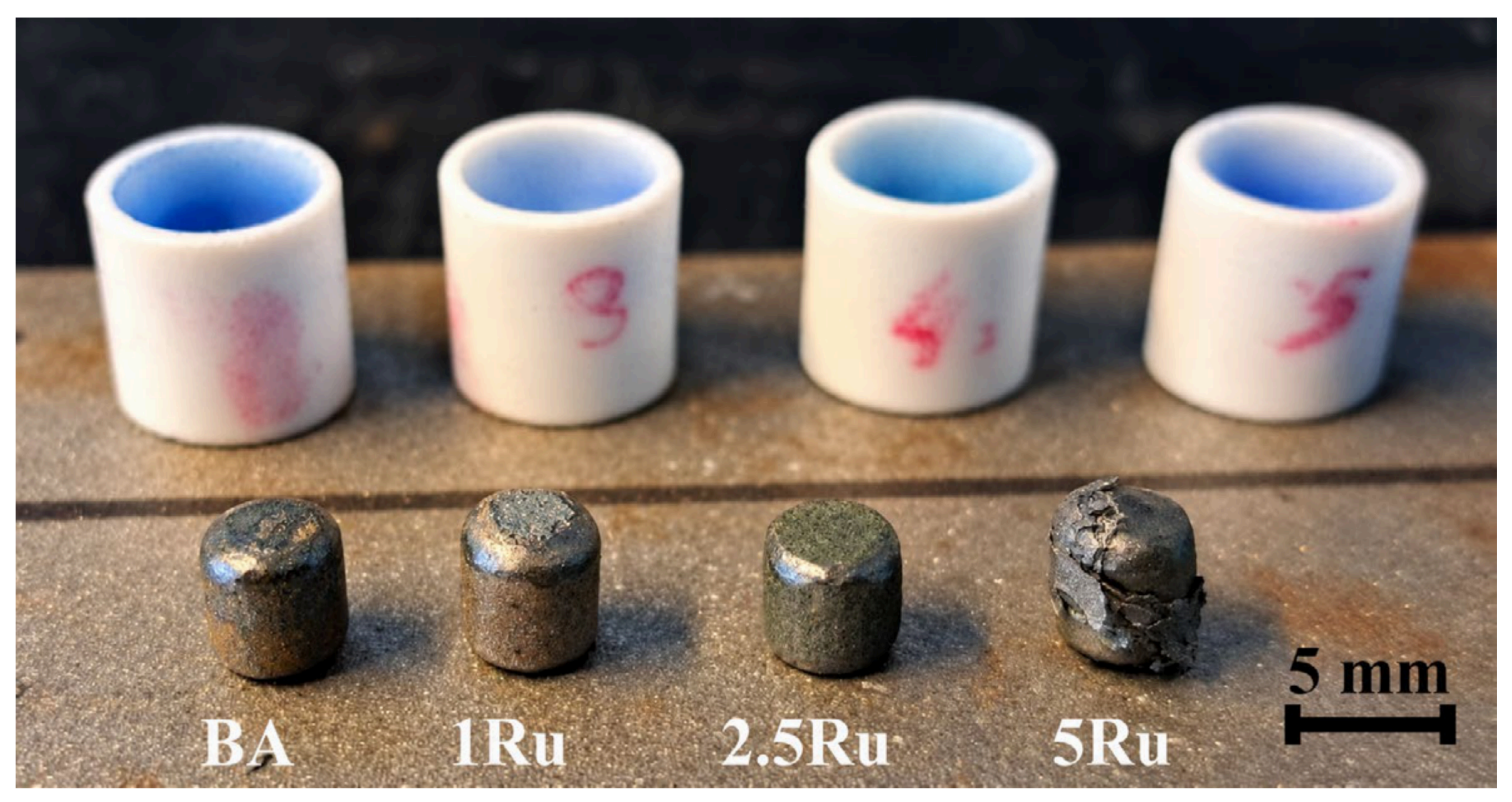
The samples after the thermogravimetric test.

Figure 7 shows the appearance of the specimens after the oxidation resistance tests. The ceramic crucibles remained intact, with no visible signs of charring, indicating that the test temperature—namely, 1100 °C—was maintained consistently throughout the experiments. Spalled oxidation products accumulated inside the crucibles; therefore, the specimen weight-gain plots showed no discontinuities or sharp transitions. The inner surfaces of the crucibles exhibited a bluish-violet color, apparently associated with the diffusion and deposition of oxidation products from the specimens, particularly those containing titanium, tungsten, and cobalt. The oxidation products of the other alloy constituents were black or gray.

All initially cylindrical specimens (Figure 1) underwent slight shape changes: the junction between the cylinder bases and the lateral surfaces became rounded and spherical, as if the cylinder edges had been chamfered. Surface darkening and the loss of mirror-like gloss indicate substantial oxidation. The BA specimen showed the least external damage. No significant spallation of the oxide scale was observed, suggesting the lowest oxidation intensity. Along the “chamfered” region, the 1Ru specimen showed noticeable oxide layer spallation; this region was light-colored and exhibited a characteristic metallic luster. The rest of its surface looked similar to the BA specimen. The 2.5Ru specimen showed even more pronounced oxidation. Nevertheless, it retained its cylindrical shape, and its overall appearance was similar to the preceding specimens. The 5Ru specimen’s surface was severely oxidized, with extensive scale spallation across its entire surface. The initially flat base of the cylinder had practically transformed into a cone due to expansion of the nominal “chamfer”, the junction between the cylinder base and its lateral surface. Thus, visual examination suggests that increasing the ruthenium content in the alloy accelerates its oxidation. A Ru content of 5 wt.% appears to be critically high, drastically reducing the alloy’s oxidation resistance.

**Figure 8:**
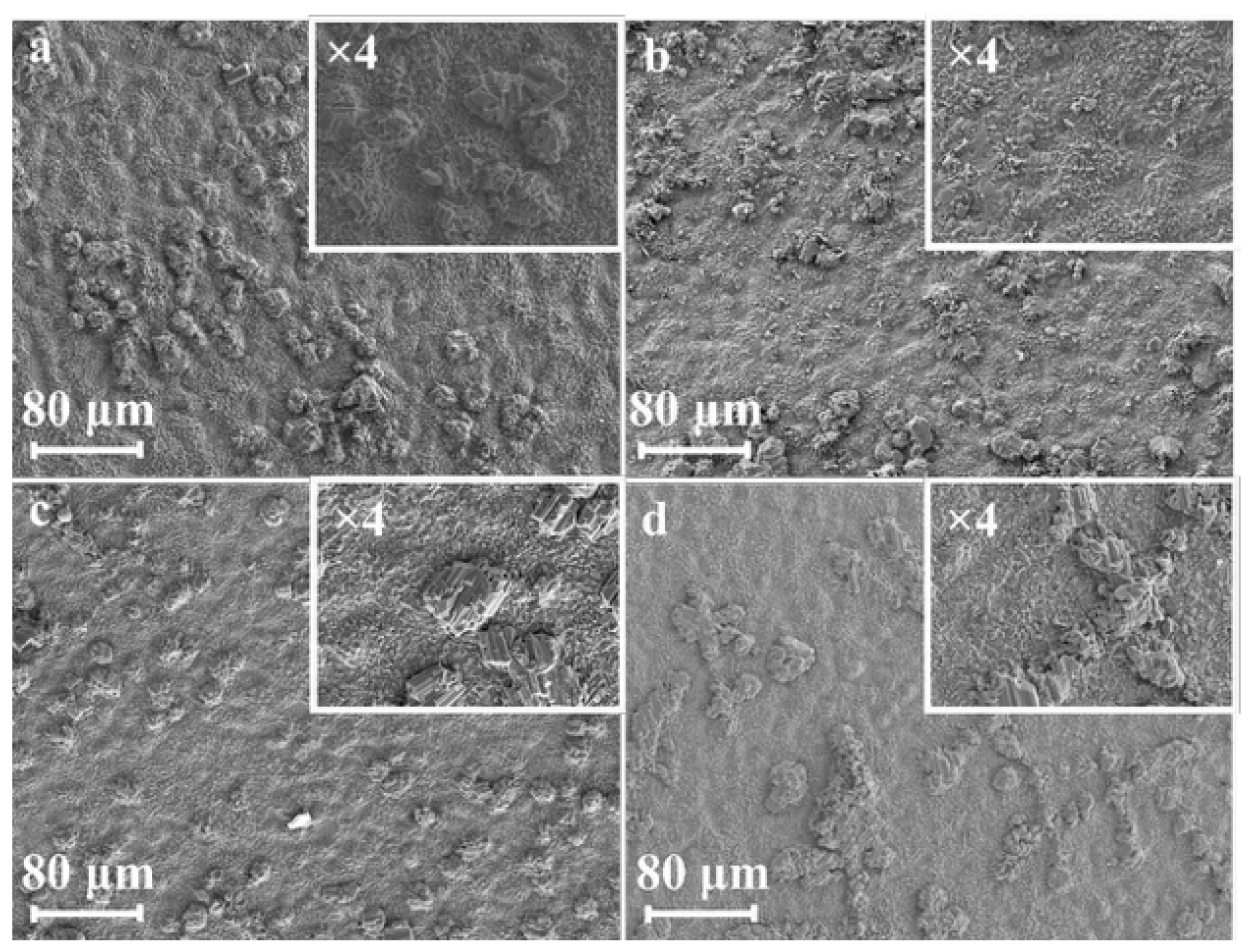
Surface morphology of the thermally exposed, unworn central regions of the friction specimens, located outside the wear tracks, for the BA (**a**), 1Ru (**b**), 2.5Ru (**c**), and 5Ru (**d**) alloys.

Figure 8 shows the surface microstructure of the alloys after oxidation resistance testing. The annealing temperature was 1100 °C, and the holding time was 100 h. Convex structures measuring 5–40 µm were visible on the surfaces of all the alloys. Their shape, size, and distribution are consistent with those of eutectic carbides, which are an integral part of the alloy microstructure. However, local SEM-EDS analysis could not confirm this interpretation. This morphology suggests that the carbides have lower oxidation resistance than the surrounding matrix material. In fact, the formation of these convex features indicates that a considerably larger amount of oxide formed over the same period. Their size correlates with the carbide size (Table 4): the carbides were finest in the 1Ru alloy and became coarser in the 2.5Ru and 5Ru alloys. The matrix also contained significant amounts of Ru and Re. These elements may form volatile oxides, reducing oxide growth.

The angular shapes in the enlarged views likely preserve the original TiC grain morphology. The more rounded and swollen regions likely result from oxidation and volume expansion of the carbide and the surrounding oxide scale. At 1100 °C, O penetrates the material’s interior along surface defects and the solid-solution/carbide interface. It reaches TiC particles located near the surface. As they oxidize, Ti, Re, and W form oxides (the other two elements are in the carbide structure; see Figure 5). These oxides occupy more volume than the former carbide, producing outward swelling or bulging. Continued oxidation causes roughening, cracking, local delamination, and the formation of a textured oxide mound. At the same time, evaporated carbon oxides within the sample may produce internal porosity beneath the protective oxide layer.

**Figure 9:**
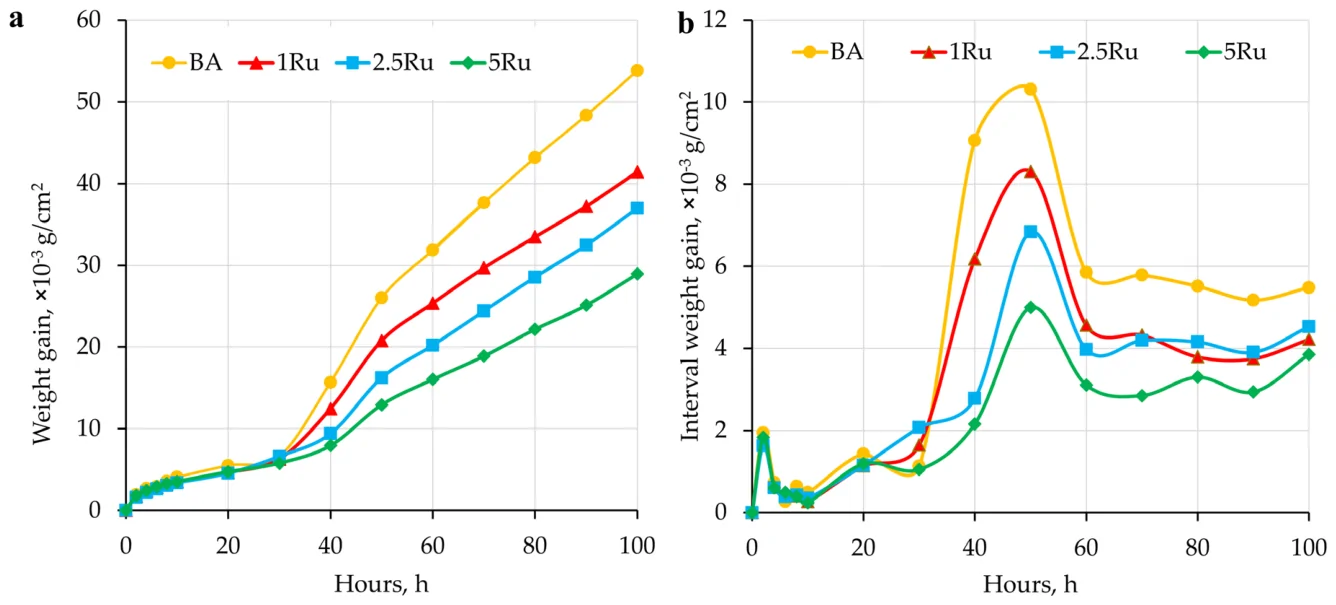
The results of the oxidation resistance tests: weight gain of the tested samples (**a**) and interval weight gain, for every 10 h of test (**b**).

Figure 9 presents the oxidation resistance test results. The samples showed different weight gains that were directly correlated with Ru content. Specifically, the highest weight gain (Figure 9a) was for the base alloy BA—54 × 10^−3^ g/cm^2^, while 1Ru—41.5 (23% lower), 2.5Ru—37 (31% lower), and 5Ru—28.8 (46% lower). Thus, increasing Ru content decreases the weight gain of the samples. As reported in the literature, Ru and Re form volatile oxides that escape the surface. During oxidation, TiC forms stable titanium oxides and carbon oxides, which also evaporate. Thus, two processes run simultaneously: weight gain from the formation of Co, Cr, Al, and Ti oxides, and weight loss from the evaporation of Ru, Re, and C oxides. For this reason, thermogravimetric data alone cannot reliably determine oxidation resistance. We need to examine the cross-sectional morphology of the oxidation samples to determine oxide layer thickness and the extent of internal oxidation. Together with external sample observation and oxidation kinetics, this will provide a more reliable estimation of the alloy’s oxidation resistance.

Figure 9b shows the weight gain of the samples during each 10-h period. Initially, oxidation proceeds quickly because the surface is clean and unprotected. During the first 2–4 h, all samples gained nearly the same weight (about 2 × 10^−3^ g/cm^2^), then it dropped to less than 0.5 and stayed in this range through the end of the first 10 h. During the first 30 h, the oxidation rate was low and nearly equal across all samples. Later, between 30 and 50 h, it increased by orders of magnitude for sample BA. Here, weight gain was more pronounced than weight loss, which may be reflected in the sample’s weight increase, but weight loss became more intense. Later, the oxidation rate stabilized and remained constant until the end of the test.

#### 3.5.2. Cross-Sectional Morphology Analyses and Oxide Scale Measurements

**Figure 10:**
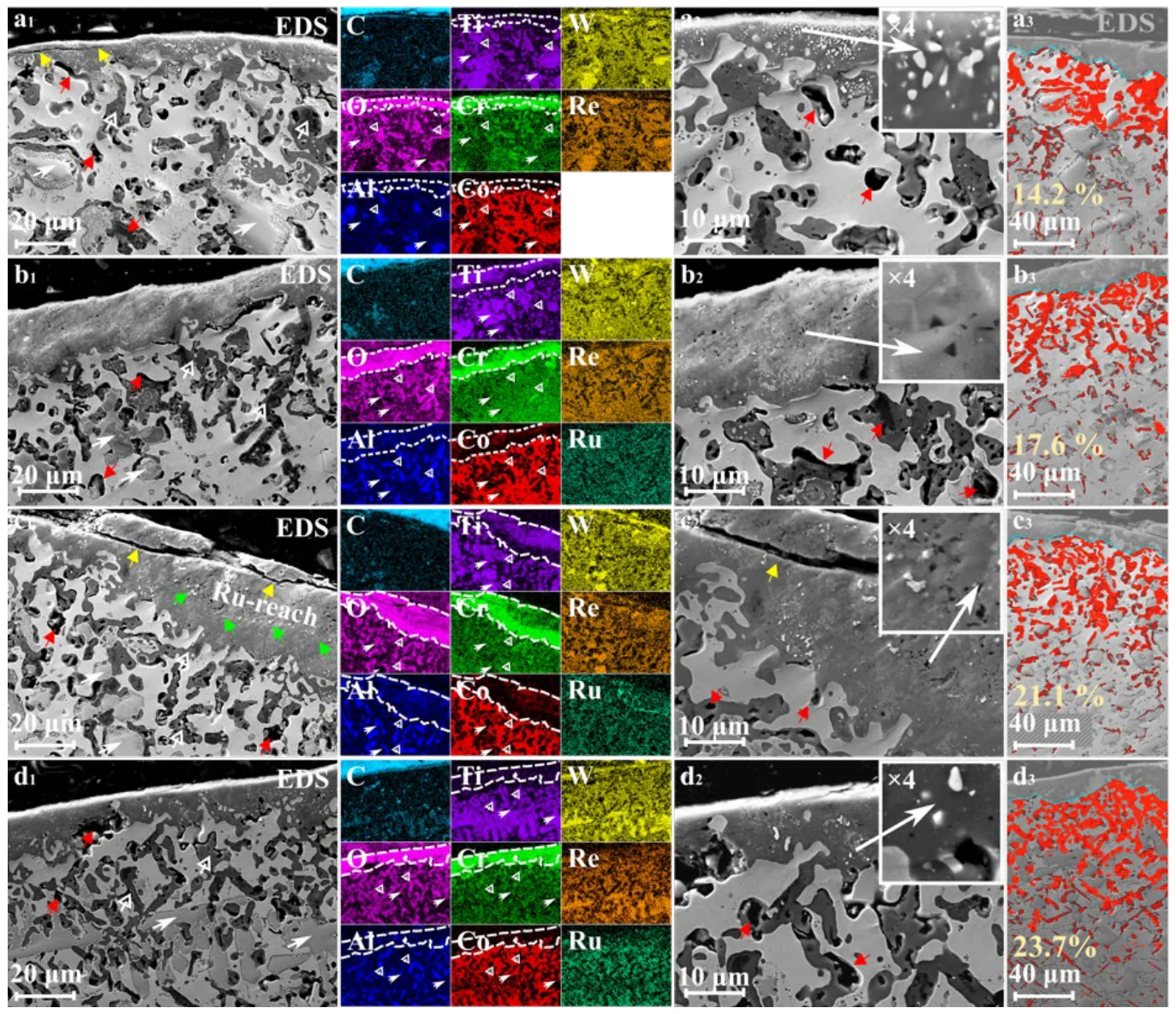
SEM micrographs showing the cross-sectional morphology of the tested samples after a 100-h oxidation resistance test (**a_1_**–**d_1_**,**a_2_**–**d_2_**), the corresponding EDS elemental maps, and representative images showing highlighted internal oxidation pockets (**a_3_**–**d_3_**).

Figure 10 shows cross-sections of the samples tested for oxidation resistance. In the EDS maps, the white lines indicate the upper and lower boundaries of the visible oxide layer. Solid arrows indicate carbides, hollow arrows indicate dark regions beneath the oxide layer, and yellow arrows indicate exfoliation cracks within the oxide layer. White dashed lines were added to facilitate interpretation of the elemental distribution maps. Red areas in Figure 10(a_3_–d_3_) are internal oxidation pockets.

An oxide layer formed on all investigated samples, with a thickness ranging from 10–35 μm for the base alloy BA (Figure 10(a_1_,a_2_)) to 15–62 μm for the 2.5Ru alloy (Figure 10(d_1_,d_2_)). Beneath the oxide layer, all samples showed a porous zone formed by the evaporation of volatile oxides and internal oxidation.

The ruthenium-free alloy BA is shown in Figure 10(a_1_–a_3_) with the corresponding EDS maps. In this sample, the visible oxide layer was 10–35 μm thick, averaging 22 μm. The hollow arrows indicate an elongated void forming along the alloy–scale interface and penetrating into the alloy, and red arrows indicate subscale porosity and cracks. The morphology of these pores does not correspond to the distribution of any alloying element or to the size and shape of the carbides (solid arrows, Figure 10(a_1_–d_1_)). Therefore, they were formed through internal oxidation and the redistribution of alloying elements, particularly those that form volatile oxides. The oxide layer (see Figure 10(a_1_) and the insert) contains numerous light spots, assumed to be unoxidized metal remnants, and no visible pores.

The 1Ru alloy formed a substantially thicker oxide scale. Unlike the BA alloy, it contained fewer bright inclusions (Figure 10(b_2_) and the enlarged insert), evidently remnants of incompletely oxidized alloy matrix or carbides. Here, some black spots also appeared, evidently due to the evaporation of volatile oxides. As in the previous alloy, an elongated void was visible along the metal–scale interface (Figure 10(b_1_), red arrow). This is even clearer in Figure 10(b_2_). This is not a crack along the phase boundary, but a cavity formed due to the evaporation of oxides just below the scale. In the Figure 10(b_2_) insert, we observed increasing porosity in the oxide scale and a few bright spots within it. We assumed these spots are the same in nature for all materials: unoxidized alloy constituents.

The oxide scale thickness was 22–48 μm, which was greater than that observed in the preceding BA specimen. The average oxide thickness was 36 μm. Two regions can be distinguished within the oxide scale (see the maps in Figure 10(b_1_)): the upper region was dominated by aluminum, cobalt, and titanium oxides, whereas the lower region was enriched in chromium oxides. Tungsten and rhenium concentrations increased below the scale’s lower boundary. Ruthenium distribution appeared visually uniform and was similar in both the alloy and the oxide scale.

On the surface of the 2.5Ru alloy, the thickest oxide scale reached 41 μm (Figure 10(c_1_,c_2_)), with a max. of 62 μm and a min. of 15 μm. The yellow arrows indicate cracks along the scale, suggesting more extensive scale spallation than in the previous specimen. The number of subsurface pores also increased compared with the preceding materials.

As in the 1Ru alloy, the oxide scale consisted of two layers: an upper layer formed predominantly by cobalt, aluminum, and titanium oxides (see the elemental maps in Figure 10(c_1_)), and a lower layer composed mainly of chromium and aluminum oxides. Interestingly, an intermediate region with high ruthenium concentration occurred between these two layers (Figure 10(c_1_), green arrows). Its high ruthenium content promoted more intense evaporation. It diffused from the specimen interior into the oxide scale and formed volatile oxides. During cooling, these oxides transformed into more stable forms and remained within the oxide scale as a layer visible in Figure 10(c_1_).

As shown in Figure 10(d_1_–d_3_), the 5Ru specimen was the most severely oxidized, as evidenced by the large amount of spalled oxide. During resin impregnation and polishing, these oxides detached spontaneously (see Figure 7). Therefore, Figure 10(d_1_,d_2_) shows a fresh oxide layer that had not yet begun to spall and had retained its integrity. Consequently, several pores are visible (see red arrows, Figure 10(d_1_)), and no cracks within the scale. The measured oxide layer thickness was 12–39 μm, with an average of 29 μm. The insert in Figure 10(d_2_) shows increasing pore size within the scale.

For the 5Ru alloy, the upper oxide layer consisted predominantly of chromium and aluminum oxides, with minor amounts of rhenium, tungsten, and ruthenium oxides. Ruthenium distribution was the same as in the 1Ru alloy: uniform throughout both the matrix and the oxide layer.

Figure 10(a_3_–d_3_) highlights the internal oxidation pockets and their area fractions within the substrate beneath the external oxide scale. The base alloy showed the lowest internal oxidation area fraction (14.2%), followed by the 1Ru alloy (17.6%). These observations suggest that even a small Ru addition promotes oxide volatilization and pore formation, increasing internal oxidation and the development of broader oxide-filled cavities. Increasing the Ru content to 2.5 and 5 wt.% further intensified subsurface oxidation, with the corresponding area fractions increasing to 21.1% and 23.7%, respectively (Figure 10(c_3_,d_3_)). Accordingly, we evaluated the oxidation resistance of Ru-containing alloys through an integrated assessment that combines mass-change curves with comprehensive subsurface microstructural characterization.

BA and 1Ru showed the highest oxidation resistance among the investigated alloys, whereas 2.5Ru and 5Ru underwent the most extensive oxidation damage. Relative to BA, the internal oxidation area fraction increased from 14.2% to 17.6%, 21.1%, and 23.7% for 1Ru, 2.5Ru, and 5Ru, respectively, corresponding to relative increases of approximately 24%, 49%, and 67%. Meanwhile, the average retained oxide-scale thickness increased from 22 µm for BA to 36, 41, and 29 µm, representing increases of approximately 64%, 86%, and 32%, respectively.

#### 3.5.3. Oxidation Mechanisms

Subsurface porosity in oxide scales can result from the intensive evaporation of higher oxides at temperatures above 1000 °C. In particular, Re_2_O_7_ is highly volatile and evaporates rapidly, reducing its amount within the oxide scales and contributing to their degradation. Upon cooling, such as during microscopic examination, it decomposes into lower oxides and may therefore not be detected using conventional analytical methods [44,45].

Ruthenium is another element that forms volatile oxides. Previous studies, together with more recent data, indicate the formation and evaporation of several higher ruthenium oxides. As with rhenium, this process contributes to the deterioration of oxide scales [46,47,48]. Tungsten also belongs to this group, although to a lesser extent. Above 1000 °C, it forms WO_3_ and other gaseous W-O species, which reduce the tungsten content in the oxide scale [49,50].

Oxygen diffusion into the alloys along phase boundaries—specifically, the solid-solution/carbide interfaces—leads to internal oxidation. As a result, W, Re, and Ru form volatile oxides. The combined content of these elements in the alloy, for example, in the 5Ru alloy, is 17% (Table 1). Internal oxidation of these elements, as well as the carbide grains, forms internal pores, which were observed in all alloys in Figure 10. These voids promote detachment of the oxide layer. Cracks were also present along the surface within the oxide layer itself (yellow arrows).

The distribution of elements within the oxide layer was non-uniform, with oxygen, chromium, and titanium predominating. Cobalt oxides were located in the lower part of the scale, whereas tungsten oxides were found in the upper part, possibly because they tend to diffuse toward the surface and evaporate. Beneath the oxide layer, the distributions of Ti, Re, and W matched those in the alloy in its initial condition. Thus, despite prolonged exposure to high temperatures, the carbide size and composition remained unchanged.

In all the investigated materials, titanium, carbon, rhenium, and tungsten exhibited similar distributions and formed a doped titanium carbide. Ruthenium did not participate in carbide formation. The internal dark regions consisted predominantly of aluminum and chromium oxides. The pores within these regions resulted from the evaporation of volatile rhenium and ruthenium oxides, and to some extent, carbon.

Based on the analysis of the oxide-scale structure, the condition of the specimens, and the specimen weight-gain curves (Figure 7, Figure 8, Figure 9 and Figure 10), the following oxidation mechanism of the alloys can be proposed:

1. Rapid oxidation of the polished alloy surface occurs during the first 2–4 h of testing, accompanied by an increase in the mass-gain rate to approximately 2 × 10^−3^ g/cm^2^. The oxide scale protects the alloy by isolating it from the external environment; consequently, the oxidation rate remains minimal for the remainder of the test, as shown in Figure 9. Cobalt, chromium, and titanium oxides form and cover the surface. Mass gain and mass loss processes occur simultaneously and compete with each other;
2. Subsequently, oxygen penetrates the alloy along the metal-carbide interfaces, initiating the oxidation of carbides, ruthenium, rhenium, and other alloying elements. Ruthenium, rhenium, and carbon oxides volatilize, forming the first internal voids in the specimens. Convex regions of titanium oxides develop on the surface (Figure 8); these continue to disrupt the protective oxide scale, thereby facilitating oxygen penetration into the internal cavities of the specimens. Mass gain and mass-loss processes occur simultaneously and compete.
3. Titanium oxides penetrate the protective oxide scale, causing its breakdown and loss of its barrier function. A large amount of oxygen reaches the cavities beneath the oxide layer. This results in a sudden, substantial increase in the weight of all specimens (Figure 9b): the internal cavity surfaces become oxidized, forming chromium and aluminum oxides within the cavities, as well as oxides of other solid-solution constituents. The significant increase in contact area due to the subsurface pores sharply intensifies oxidation and the volatilization of rhenium and ruthenium. Ruthenium volatilizes from the alloys faster than rhenium, although all alloys contained 6 wt.% Re. This creates a paradox: the most severely damaged specimen, 5Ru, showed the lowest mass-gain rate, precisely because rhenium oxides volatilize.

Therefore, adding ruthenium decreases the oxidation resistance of these alloys; however, only a comprehensive analysis of the surface and subsurface microstructure can establish this conclusively. Conventional weight gain versus time plots provide misleading results for these alloys. In terms of oxidation resistance, the alloys rank as follows: BA → 1Ru → 2.5Ru → 5Ru, where BA denotes the base alloy, and 5Ru has the highest ruthenium content.

### 3.6. Tribological Performance

#### 3.6.1. Wear Tests

**Figure 11:**
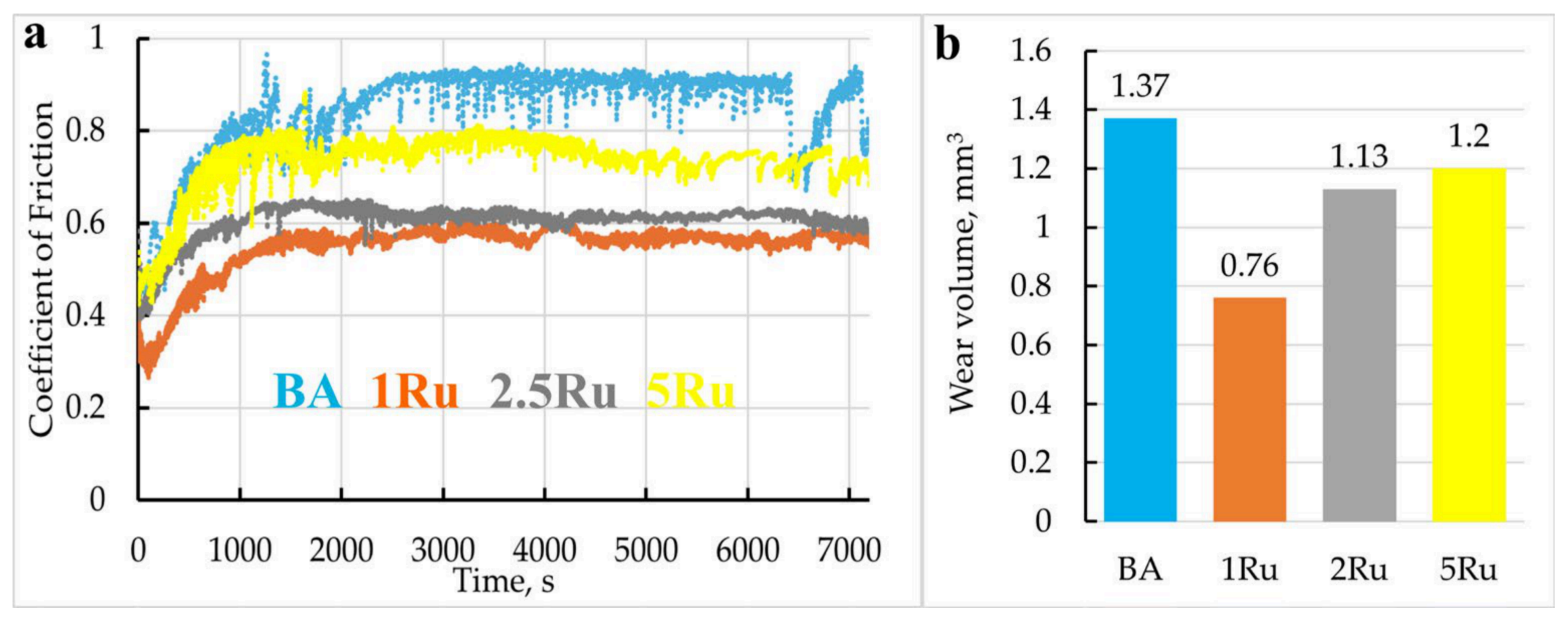
The graphs of coefficient of friction (**a**) and wear volume data (**b**) for the tested materials.

Figure 11 shows the tribological test results. The average CoF for the samples were: 0.848, 0.548, 0.605 and 0.736 for BA, 1Ru, 2.5Ru and 5Ru alloys. BA → 5Ru → 2.5Ru → 1Ru. The CoF of the most efficient sample 1Ru was 35% lower than that of the base material BA.

The best tribological behavior of 1Ru results from an optimal combination of a strengthened matrix (Figure 6), refined carbides (Figure 4, Table 4), and increased carbide hardness (Figure 9a). For 2.5Ru, the coefficient of friction was 27%, and for 5Ru, it was 13% lower than for BA. This increased hardness and larger carbide grain size may be the main reason for the high CoF. For BA, the matrix was the softest, which, together with hard carbides, resulted in the highest CoF values among the materials studied. For alloys 1Ru and 2.5Ru, the lowest CoF values may be explained by more intensive formation of TiO_2_ (see Ti and O EDS maps, Figure 10), which may act as a solid lubricant.

All CoF graphs showed a distinct transition period, during which it increased, about 800–900 s for BA and 5Ru, and 1200–1300 s for the other 2 materials. For 1Ru, after the running-in period, the graph was smooth and uniform despite some oscillations at 2200, 4000, and 6500 s. After the initial period, the 2.5Ru curve was the most uniform. The 5Ru curve showed persistent oscillations. In the final stage, it showed a decreasing trend but an increasing oscillation amplitude. The BA graph was less stable and suffered from the most substantial oscillations. Especially in the final stage, this may indicate friction-surface failure.

To summarize, the 1Ru sample had the lowest and most stable CoF, while BA had the highest average CoF and the least stable CoF graph.

Figure 11b presents the volumetric wear values of the investigated alloys. The base alloy (BA) showed the highest wear (1.37 mm^3^). The 1Ru alloy showed the lowest wear (0.76 mm^3^), a 0.61 mm^3^ (44.5%) reduction relative to BA. For the 2.5Ru alloy, wear decreased by 0.24 mm^3^ (17.5%), whereas for the 5Ru alloy, it decreased by only 0.17 mm^3^ (12.4%). Thus, under the conditions of the present experiment, the alloy containing 1% Ru exhibited the highest wear resistance, whereas the base alloy demonstrated the lowest wear resistance. As in the CoF graph (Figure 11a), alloys 1Ru and 2.5Ru showed the lowest wear, which may also be attributed to higher TiO_2_ content in the triboinduced layer (Figure 10, Table 6).

#### 3.6.2. Friction Surface 3D Topography

**Figure 12:**
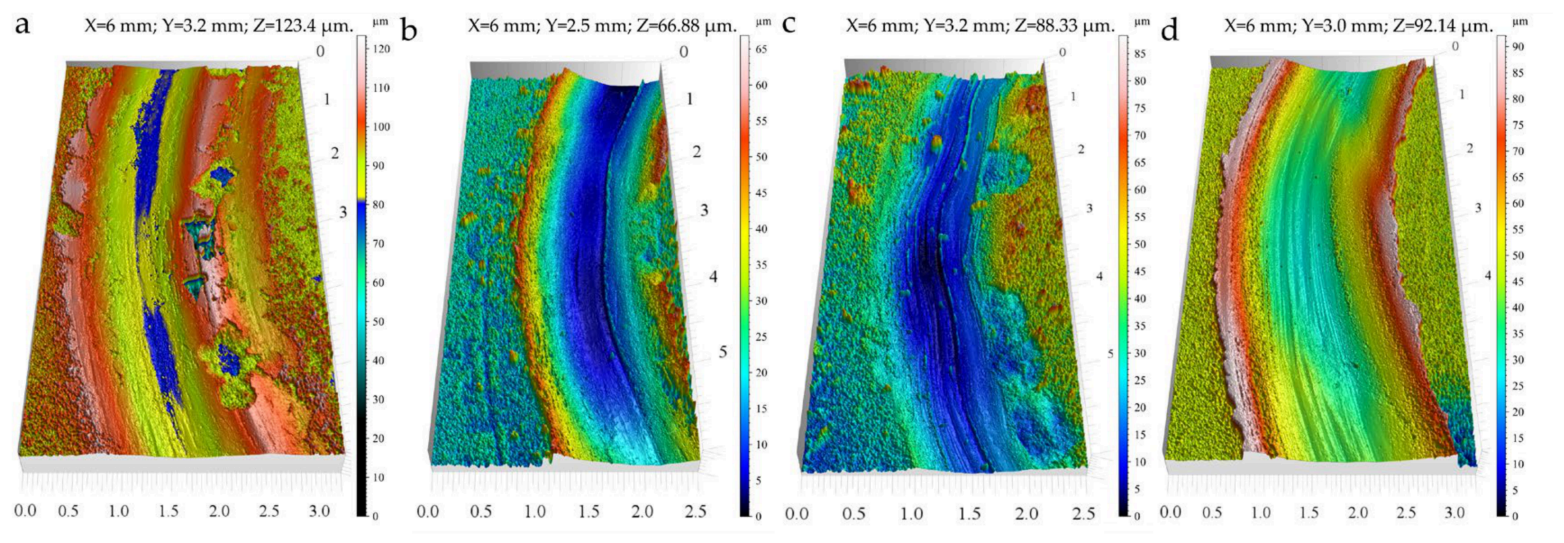
3D surface topographies of wear-track segments for the BA (**a**), 1Ru (**b**), 2.5Ru (**c**), and 5Ru (**d**) alloys. The color scale bar beside each panel represents surface height in micrometers.

Figure 12 shows the morphology of the wear scars. The base alloy BA exhibited the most severe damage to the worn surface, including the most pronounced local depressions and abrupt height variations of 20–30 μm. The maximum wear-track depth was 123.4 μm (Figure 12a), while the wear-track width ranged from 1 to 1.2 mm (Figure 12a). Delamination of the triboinduced secondary structures and longitudinal abrasive grooves produced by detached titanium carbide particles sliding through the soft matrix were observed, along with a deep, extended depression in the central part of the wear track. Along the track sides, spallation of the secondary structures and accumulation of wear debris were evident.

The 1Ru alloy wear track was the most uniform. Its central region gradually deepens, reaching 66–67 μm, while the track width was 0.9–1.0 mm. The groove contained several abrasive scratches, whereas its edges showed no signs of delamination of the secondary structures.

As the ruthenium content increased further, the wear track of the 2.5Ru alloy became wavy and heterogeneous. Pronounced deep longitudinal grooves and ridges were observed, along with signs of delamination of the triboinduced layers at the edges. The wear-track width was 1.0–1.1 mm, and its depth as approximately 88 μm. The wear track of the 5Ru alloy was wide, measuring 1.3–1.4 mm, and showed significant deformation, manifested as an inward deflection toward the specimen. The depression depth was approximately 92 μm, which was the greatest among the ruthenium-containing alloys. Its surface showed pronounced deformation features, including high lateral pile-ups and longitudinal grooves.

Thus, the 1Ru alloy exhibited the lowest wear, as its wear track was the narrowest and least damaged. Adding ruthenium improved the base alloy’s wear resistance, with an optimum concentration of 1% Ru. Further additions of ruthenium reduced the alloy’s wear resistance and are therefore not advantageous.

#### 3.6.3. Wear Surface SEM Examinations

**Figure 13:**
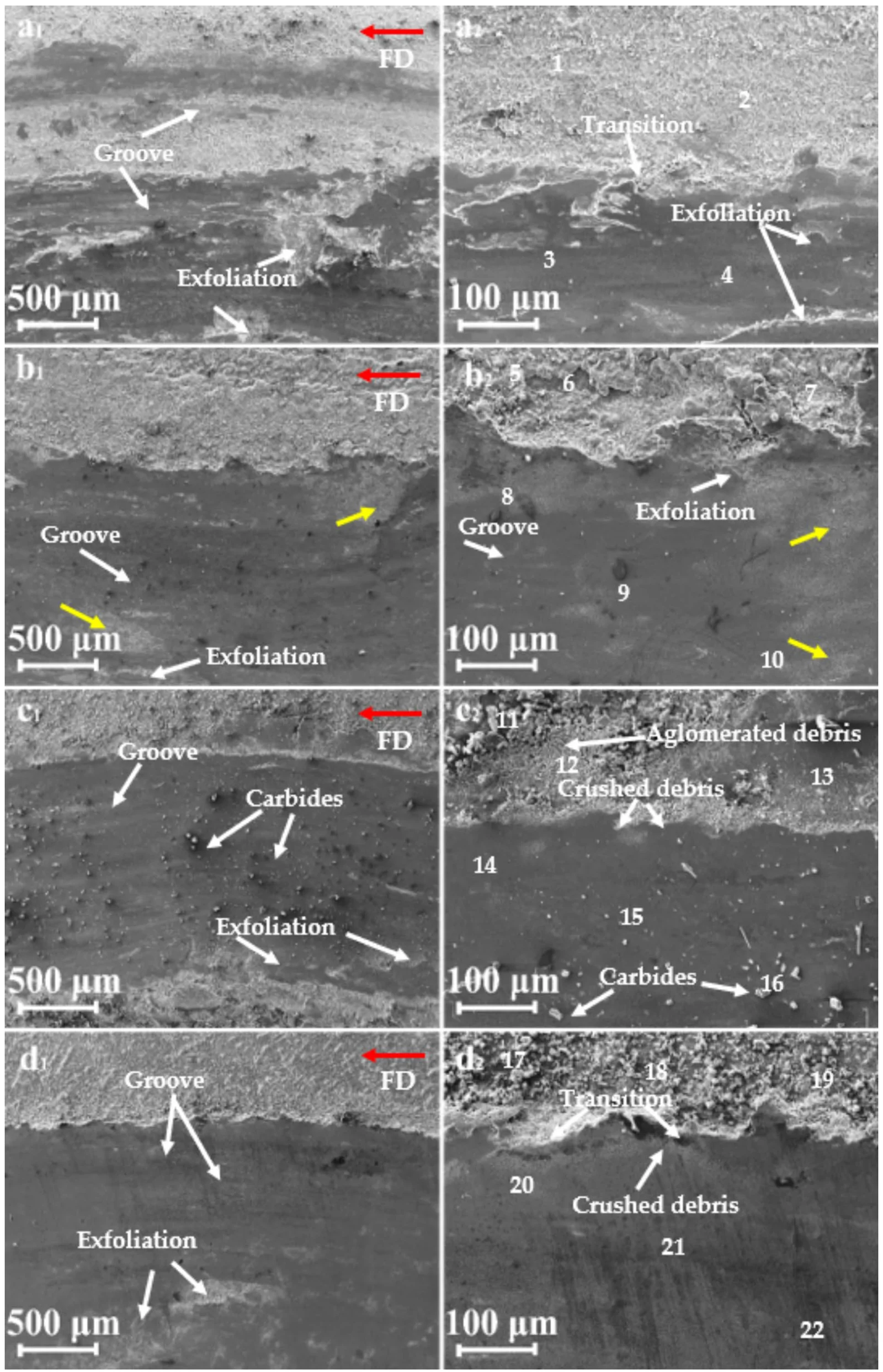
SEM micrographs of the wear tracks of the base alloy BA (**a_1_**,**a_2_**) and the 1Ru (**b_1_**,**b_2_**), 2.5Ru (**c_1_**,**c_2_**), and 5Ru (**d_1_**,**d_2_**) alloys. FD indicates the sliding direction; numbered locations 1–22 identify the positions of the EDS point analyses reported in Table 6. FD—Friction Direction.

Figure 13 presents the topography of the friction surfaces of the investigated alloys. The upper part of the image shows the undamaged (thermally exposed) surface, which was not involved in the friction process, whereas the lower part corresponds to the friction track. The numbers indicate the locations where point spectra were collected to determine the elemental composition of the friction surfaces. Table 6 presents the EDS results. The friction surface of the base alloy is shown in Figure 13(a_1_,a_2_). The thermally exposed area is relatively smooth and contains distinct regions with different contrast, indicating a non-uniform oxide layer, likely due in part to the preferential oxidation of titanium carbides [51].

**Table 6:** The results of point-EDS scanning of the friction surfaces of the tested samples from Figure 13 (1–4: BA; 5–10: 1Ru; 11–16: 2.5Ru, 17–22: 5Ru). Bold style—for the triboinduced layer.

Scan Point	Element Content, wt.%									
	C	O	Al	Si	Ti	Cr	Co	W	Re	Ru
1	3.87	21.56	0.18	0.08	13.3	13.3	46.28	1.06	0.37	0
2	4.16	21.22	0.13	0.11	12.27	12.39	48.37	0.93	0.42	0
**3**	**4.73**	**17.21**	**2.63**	**1.02**	**10.8**	**12.95**	**49.38**	**1.17**	**0.11**	**0**
**4**	**5.88**	**18.95**	**1.84**	**0.87**	**10.88**	**11.7**	**47.8**	**1.89**	**0.19**	**0**
5	1.57	22.87	0.28	0.03	14.58	19.27	38.48	2.34	0.29	0.29
6	1.91	19.87	1.3	0.01	15.59	17.47	40.69	2.48	0.37	0.31
7	1.15	20.39	1.65	0.06	16.27	18.9	39.19	1.71	0.31	0.37
**8**	**2.7**	**22.73**	**1.27**	**0.01**	**12.97**	**9.33**	**48.47**	**2.29**	**0.12**	**0.11**
**9**	**2.57**	**20.52**	**2.13**	**0.11**	**12.36**	**8.87**	**50.76**	**2.5**	**0.09**	**0.09**
**10**	**2.58**	**21.38**	**0.88**	**0.88**	**11.67**	**9.81**	**51.1**	**1.58**	**0.11**	**0.01**
11	2.14	17.41	1.98	0.01	13.3	19.72	43.68	1.11	0.23	0.42
12	1.36	19.28	1.46	0.01	9.23	20.08	46.05	1.75	0.21	0.57
13	2.21	23.37	0.87	0.08	12.03	21.06	37.4	2.21	0.17	0.6
**14**	**3.95**	**24.19**	**1.34**	**0.27**	**16.89**	**11.01**	**39.93**	**2.11**	**0.21**	**0.1**
**15**	**2.55**	**21.33**	**3.82**	**0.32**	**18.94**	**10.9**	**40.3**	**1.69**	**0.07**	**0.08**
**16**	**3.21**	**23.03**	**1.9**	**0.26**	**16.15**	**12.31**	**41.31**	**1.02**	**0.08**	**0.73**
17	4.2	22.08	2.32	0.07	4.71	24.04	38.59	1.98	0.48	1.53
18	4.05	19.72	1.6	0.12	7.63	24.93	37.15	2.54	0.37	1.89
19	3.81	21.76	0.9	0.08	6.91	26.43	35.5	2.01	0.39	2.21
**20**	**2.88**	**31.12**	**0.59**	**0.81**	**14.21**	**26.29**	**22**	**1.25**	**0.18**	**0.67**
**21**	**3.62**	**32.79**	**1.26**	**0.92**	**13.78**	**24.29**	**19.58**	**3.21**	**0.13**	**0.42**
**22**	**3.22**	**34.44**	**0.64**	**1.05**	**12.32**	**32.31**	**23.15**	**2.14**	**0.22**	**0.51**

The BA alloy friction surface was also non-uniform, with pronounced delamination and deep surface grooves throughout the friction track (Figure 13(a_1_)). These features indicate that the wear process involves a combination of oxidation-exfoliation and abrasive wear mechanisms. The transition between the thermally oxidized region and the triboinduced layer was relatively sharp, although locally disrupted (Figure 13(a_2_)).

Figure 13(b_1_,b_2_) shows the friction surface of the alloy 1Ru. The thermally induced layer was rougher than in the previous case, and the transition was steeper, which may indicate faster oxidation and deeper oxygen penetration. The light areas of the surface (yellow arrows, Figure 13(b_1_,b_2_) might contain heavier oxides, like tungsten and cobalt oxides. The friction surface was uniform and showed no evidence of deep grooves, exfoliation, or other major surface failures, suggesting mild abrasion as the primary wear mechanism. Some minor edge exfoliation (Figure 13(b_2_)) occurred at the transition from the friction surface to the thermally affected area and resulted from compression failure of the triboinduced layer.

Figure 13(c_1_,c_2_) shows the friction surface of the alloy 2.5Ru. Here, we note that increasing the Ru content increases the roughness of the oxidized surface, creating more oxidized debris and resulting in debris agglomerates (see Figure 13(c_2_)). The friction surface demonstrated deep grooves and exfoliation wear. We may suppose that more intensive wear is caused by carbides spalling from the friction surface. The increased debris is swept out of the wear track (Figure 13(c_2_)), creating sites where the debris agglomerates.

The thermally induced layer of the 5Ru alloy showed the greatest non-uniformity compared with the other alloys. Here, carbide oxidation and internal oxidation (see Figure 10(d_1_,d_2_)) reduce the surface strength, and consequently, cause deformation under ball sliding (Figure 12d). The triboinduced layer of this sample is relatively uniform, but contains many grooves and exfoliations, indicating oxidation–exfoliation wear. The triboinduced layer structure in Figure 13(c_2_) appears brittle, which may lead to more severe wear. Internal oxidation created subsurface porosity (Figure 10(d_1_,d_2_)), reducing load-carrying capacity, causing severe wear-track deformation, and increasing measured volume wear.

The analyses in Table 6 provide several observations. First, in the thermally exposed layer, the Cr content increases with Ru content, namely, about 12.9 wt.% for BA; 1Ru: 18.6 wt.%; 2.5Ru: 20.3 wt.% and for 5Ru: 25.1 wt.%. Thus, as the Ru content in the alloy increases, the local Cr concentration in the outer oxidized zone increases. This is especially evident for 5Ru, where on pp. 17–19, the Cr content was 24.04–26.43 wt.%. In the triboinduced layer, the Cr content decreased for samples: 12.33 wt.%. for the base alloy, 9.3 wt.% for the 1Ru alloy, 11.4 wt.% for the 2.5 Ru alloy, and 14.3 wt.% for 5Ru. In the 5Ru alloy, the chromium distribution differed. Although its average content in the tribolayer was higher than in the surrounding areas, it remained the highest among all the investigated materials, reaching a maximum value of 32.31 wt.%. This suggests possible local chromium enrichment in certain areas of the friction zone.

In general, Ru increases Cr content in both thermally affected and triboinduced layers. This may be explained by the continuous removal of Cr-oxide debris from the wear surface and its collection outside the wear track. Other elements also introduce their oxides into the triboinduced layer, replacing and altering the sites of Cr oxidation and collection.

The same behavior was observed for Al—another important element to form the protective oxide scale in cobalt superalloys: its content in the thermally affected area is lower than for triboinduced layer. The increase in silicon content on the wear tracks is attributed to wear of the counterpart ball (Si_3_N_4_) and the accumulation of wear debris on the friction surface.

The second important element is Ti. Its content generally decreases as Ru content increases, as Ti is partially replaced by other oxides. In the thermally oxidized area, the Ti content was 12.79 wt.% for BA, 15.48 wt.% for 1Ru, 11.52 wt.% for 2.5Ru, and only 6.42 wt.% for 5Ru. The highest Ti content in the 1Ru alloy likely comes from retained TiC particles and titanium oxides on the surface.

In the triboinduced layer, the Ti content increased, particularly for the 2.5Ru and 5Ru alloys, reaching average values of 17.33 and 13.44 wt.%, respectively. This may be explained by TiC wear debris accumulation on the friction surface, oxide exfoliation, and the exposure of unoxidized TiC particles. Together with oxide debris adhesion, these processes increase the Ti content in the triboinduced layer. For 2.5Ru and 5Ru, the Ti content rose with C; we assume both are part of TiC and reflect the accumulation of abrasive particles on the friction surface.

In the 5Ru alloy, the Ti content was approximately 2 times higher than that in the surrounding areas. This high local Ti concentration may promote abrasive wear, exfoliation wear, and spallation of the triboinduced layer, as observed in Figure 12d and Figure 13(d_1_,d_2_).

In the studied alloys (Table 6), cobalt was the primary element in all tested samples. Its average content ranged from 47.32 wt.% in BA to 37.08 wt.% in 5Ru. Thus, increasing the Ru content increases Cr and Al in the triboinduced layer while decreasing Ti and Co. These changes in the chemical composition of the triboinduced layer may contribute to the observed differences in wear rate. To complement these findings, XRD analyses were performed to identify the phases that formed in the alloys and to determine their roles in friction and wear.

#### 3.6.4. Phase Composition of Triboinduced Layer

**Figure 14:**
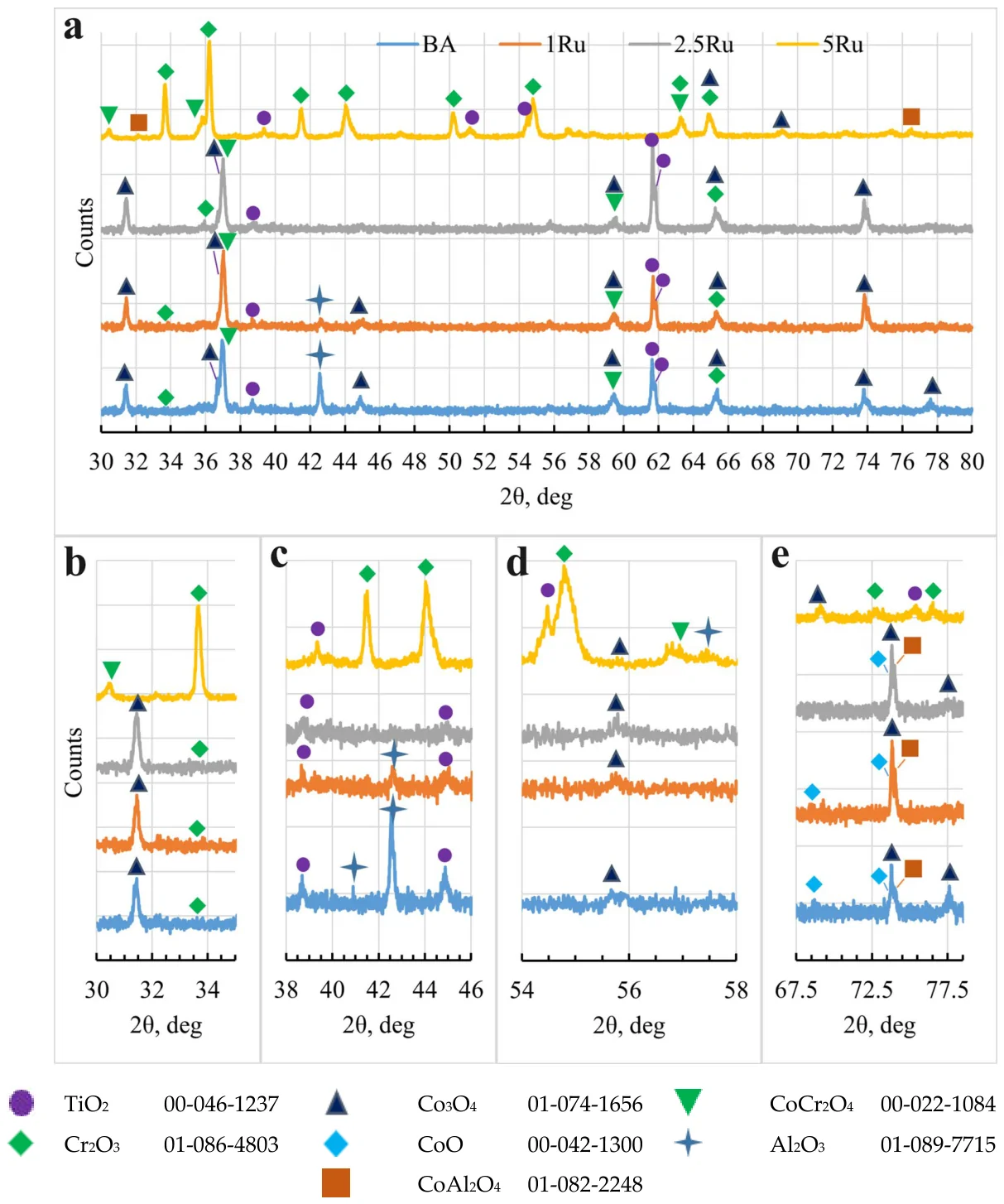
XRD pattern (**a**) and enlarged inserts (**b**–**e**) acquired from the friction surface of the samples.

Figure 14 presents the X-ray diffraction patterns of the friction surfaces. Because many components can form solid solutions and complex spinel-type oxides, separating them reliably is difficult. Therefore, we can identify only the most probable phases and those likely to be present. X-ray diffraction is also a surface-sensitive technique and generally does not detect phases located deeper than approximately 0.5–1 μm. Tungsten, rhenium, and ruthenium oxides are volatile; consequently, their concentration in the surface layer may be low and undetectable by X-ray diffraction, whereas their content at greater depths may be considerably higher.

Some phases also have very similar 2θ angles. Co_3_O_4_, CoCr_2_O_4_, and CoAl_2_O_4_ cannot be reliably distinguished because they are structurally related to Co_3_O_4_, in which cobalt is partially substituted by chromium or aluminum, and they share very similar cubic spinel structures. For example, Co_3_O_4_ has the main diffraction peaks at approximately 31.34°, 36.936°, 44.921°, 55.797°, 59.51°, 65.4° and 74.32° (PDF 01-074-1656). CoCr_2_O_4_ exhibits reflections at approximately 30.304°, 35.715°, 43.385°, 53.888°, 63.103°, and 74.665° (PDF 00-022-1084). CoAl_2_O_4_ produces a very similar series of peaks at approximately 31.182°, 36.741°, 44.680°, 55.488°, 59.521°, 65.033°, 73.9° (PDF 01-082-2248).

This study aims to determine the predominant oxide composition of the friction surfaces rather than the exact composition of the spinels and their derivatives. These phases derive primarily from the oxides of four alloying elements—cobalt, chromium, titanium, and, to a lesser extent, aluminum, which was present at approximately 2% in the alloys.

All diffractograms (Figure 14a) exhibited well-defined peaks corresponding to oxide phases. Peaks associated with the cobalt-based solid solution or titanium carbides were absent, indicating a relatively thick oxide scale. The predominant phases were Cr_2_O_3_, Co_3_O_4_, and CoO. Spinel phases and aluminum oxide were also present, but in smaller quantities. Thus, a multiphase oxide layer forms on all friction surfaces, with the balance among its constituents depending on ruthenium content, since the concentrations of all other elements and the tribological test conditions were identical.

The BA alloy, Figure 14, exhibited a series of reflections corresponding to Co_3_O_4_, Cr_2_O_3_, and CoCr_2_O_4_, together with a small number of low-intensity reflections attributable to Al_2_O_3_. The diffractograms of BA, 1Ru, and 2.5Ru predominantly contained cobalt and titanium oxides, with smaller contributions from chromium and aluminum oxides. In contrast, chromium oxides and spinel phases dominated the 5Ru alloy.

On the BA diffractogram, a large peak was particularly noticeable at approximately 37°, Figure 14a. This peak may correspond to two phases: chromium oxide (Cr_2_O_3_; PDF 01-086-4803) and the CoCr_2_O_4_ spinel (PDF 00-022-1084). The presence of separate Co_3_O_4_ oxide at this reflection is also possible, though less likely, since numerous Co_3_O_4_ reflections appear at other positions.

The dominant phases in this alloy were cobalt and chromium oxides, as well as a Co-Cr spinel. Titanium oxides were also present in significant amounts, as indicated by the strong reflection at 61.6° (Figure 14a) and several weaker reflections in the 38–46° range (Figure 14c). Figure 14b shows a reflection corresponding to Al_2_O_3_ (PDF 01-089-7715), while Figure 14e contains a reflection of CoAl_2_O_4_ (PDF 01-082-2248). Thus, the oxide layer on the friction surface of this alloy is primarily composed of chromium and cobalt oxides. Titanium oxides are incorporated into the layer as the alloy wears and the surfaces of the carbides and their wear debris progressively oxidize. Elemental mapping does not indicate significant enrichment of the oxide layer with aluminum. However, internal cavities containing considerable amounts of aluminum oxides were observed (elemental mapping, Figure 10). As the alloy wears, these oxides become exposed at the surface and progressively contribute to triboinduced structure formation.

Compared with BA, the diffractogram of 1Ru showed more pronounced TiO_2_ reflections at 61.6° (Figure 14a) and CoAl_2_O_4_ at 73.8° (Figure 14e), while the weaker Al_2_O_3_ reflection (Figure 14c) and the minor Co_3_O_4_ peaks (Figure 14a,e) were absent. The distribution of the other phases was similar to that of the BA alloy. Thus, adding 1 wt.% Ru induces some changes in phase composition; however, these changes may reflect differences in oxidation kinetics (Figure 14a,b) and wear intensity, resulting in smaller amounts of Al_2_O_3_ from the subsurface regions being exposed at the surface.

In the diffractogram of 2.5Ru, the dominant phases were Co_3_O_4_, Cr_2_O_3_, CoCr_2_O_4_, and TiO_2_, with CoO detected locally. Overall, the phase distribution was similar to that observed for the 1Ru alloy. Additional chromium oxide peaks appeared (Figure 14a, 36°), while the aluminum oxide (Figure 14c) and cobalt oxide (Figure 14e) reflections were absent. At the same time, the TiO_2_ peaks increased in intensity (Figure 14a), as did the CoAl_2_O_4_ reflections (Figure 14e). The oxide layer was less homogeneous than in the two preceding alloys.

For the 5Ru alloy, Cr_2_O_3_, Co_3_O_4_, CoO, TiO_2_, CoCr_2_O_4_, and CoAl_2_O_4_ were identified. In this material, the triboinduced layer was predominantly composed of chromium oxides, with the strongest peaks at 33.6° and 36.17° corresponding to Cr_2_O_3_. This indicates substantially different wear dynamics compared with the other investigated materials. Significant amounts of cobalt and aluminum oxides were also present (Figure 14). Another characteristic feature is the reduced amount of titanium oxides (Figure 14a,c,d) and spinel phases (Figure 14a,e). Thus, 5 wt.% Ru appears to shift the surface tribofilm from a Co-rich oxide layer toward a Cr-rich protective oxide film. This may be a key factor in explaining the changes in the friction coefficient and wear rate of 5Ru at 1100 °C.

In addition to the phases identified above, small amounts of Re, Ru, and W oxides may also be present. These oxides may remain in the subsurface regions of the triboinduced structures and remain undetectable by XRD because their diffraction reflections are low in intensity.

#### 3.6.5. Wear Mechanisms

Based on the results, wear of the base alloy was the most intense and was governed by the simultaneous occurrence of abrasive and oxidative wear, as well as exfoliation of the tribologically induced secondary structures, which exposed new titanium carbide grains and intensified the abrasive component. A high amount of aluminum oxide should also be noted (Figure 14c), as Al_2_O_3_ is well-known for its abrasive properties. The unstrengthened matrix provides insufficient support for the carbide particles and is intensively damaged by them, while the carbides themselves are prone to fragmentation and pull-out. Consequently, deep abrasive grooves are formed (Figure 13(a_1_,a_2_)). As a result, the friction surface becomes severely damaged and irregular. Due to oxide-layer thickening and cyclic loading from the counterbody, cracks develop within the oxide layer, leading to delamination, fracture, and repeated entrainment into the contact zone. Thus, the dominant wear mechanism in this alloy is a synergistic combination of matrix abrasion, carbide-assisted third-body wear, and oxidation–exfoliation wear.

The 1Ru alloy showed the lowest coefficient of friction, and its wear was 44.5% lower than the base alloy. This improved tribological performance results from combined enhancements in matrix and carbide properties, as well as changes in carbide morphology, size, and distribution. When carbide particles fragment or pull out, they produce a less pronounced abrasive effect than those in the base alloy. According to the diffraction analysis (Figure 14), cobalt oxides remained dominant in the oxide layer, while the amount of titanium oxide increased. At the same time, the elemental analysis (Table 6) indicates a substantial increase in oxygen and titanium content, accompanied by a decrease in carbon content compared with the base alloy. These results suggest that a tribolayer enriched in titanium oxides forms on the friction surface, whereas the probability of detecting titanium carbide grains decreases. The finest TiC grains observed among the studied materials may promote faster carbide oxidation and titanium oxide agglomeration on the friction surface while reducing the abrasive action of the carbides. Consequently, the titanium oxides can act as solid lubricants [52], reducing both the coefficient of friction and the wear rate (see Figure 11). The wear track (Figure 13(b_1_,b_2_)) was relatively narrow and shallow and contained only minor abrasive grooves. Therefore, the dominant wear mechanisms are mild abrasive wear and oxidative wear. Thus, adding 1 wt.% Ru appears to provide an optimum balance between matrix strengthening, carbide stabilization, and the formation of a comparatively stable, low-friction oxide layer.

The 2.5Ru alloy exhibited lower wear resistance than the 1Ru alloy and a higher coefficient of friction. Nevertheless, its wear resistance remained higher than that of the base alloy. The wear track was less uniform, with pronounced delamination of the oxide layers along its edges and a considerable number of titanium carbide grains exposed on the surface (Figure 13(c_1_,c_2_)). Scratches and grooves appeared along the wear track, indicating intensified abrasive wear caused by the larger carbide particles. The diffraction analysis results (Figure 14) revealed an increased amount of titanium oxides, which, as in the 1Ru alloy, would be expected to reduce the coefficient of friction. However, this effect was not observed for several reasons. First, we attribute this to the increased chromium oxide content, which embrittles the triboinduced layer. Second, intensive internal oxidation and the development of subsurface voids reduce the load-carrying capacity of the friction surface and increase surface deformation, carbide fragmentation, and pull-out. This increases the intensity of abrasion. Thus, the dominant wear mechanisms in this alloy are oxidative wear, abrasive wear, and exfoliation of the triboinduced layer.

The 5Ru alloy exhibited the lowest wear resistance among the ruthenium-modified alloys. X-ray phase analysis revealed a high concentration of chromium oxides and a reduced amount of titanium oxides (Figure 14), which may indicate embrittlement of the triboinduced surface structures. This embrittlement promotes cracking, fragmentation, and spallation of the oxide layer (Figure 13(d_1_,d_2_)) under repeated mechanical loading.

Another contributing factor is intensive internal oxidation, which increases the number of internal pores and weakens the subsurface material layer. Intensive internal oxidation primarily governs its wear behavior by reducing the load-carrying capacity of the friction surface. Consequently, the surface loses its ability to withstand the counterbody load and undergoes subsurface deformation, as shown by the sharp increase in friction-track depth. The surface also shows pronounced longitudinal grooves, lateral pile-ups, and extensive plastic deformation. Detached oxide fragments and loose carbide particles act as abrasive third bodies, causing repeated changes in the real contact area. Therefore, the principal wear mechanism of the 5Ru alloy involves intensive internal oxidation followed by a loss of the friction surface’s load-carrying capacity, accompanied by oxide exfoliation and carbide-caused abrasion.

Regarding the abrasive component of the wear mechanisms described above, increasing the ruthenium content from 1 to 5% simultaneously increases two characteristics: carbide hardness and carbide size. In our opinion, these changes intensify both abrasive and oxidative wear. The larger the carbide, the greater its abrasive action and the more severely it damages the friction surface. The same is true for the hardness increase. Meanwhile, the matrix microhardness remains much lower; see Figure 6a. Consequently, the damaged surface undergoes oxidation more rapidly. In addition, larger carbides, as observed in the 2.5Ru and 5Ru alloy, require more time to oxidize than finer carbides, as found in the 1Ru alloy, and therefore retain their abrasive character for a longer period. We believe this hinders the formation of a protective oxide film, increasing the coefficients of friction and wear.

## 4. Conclusions

We successfully fabricated CoCrWReAl-xRu-TiC eutectic alloys (x = 0, 1, 2.5, and 5 wt.%) by tungsten-arc induction melting. All compositions exhibited good castability, with no detectable casting porosity, cracks, or elemental segregation. Their microstructures consisted of a Co-based FCC solid solution and TiC carbides containing Re and W, whereas Ru remained in the metallic matrix without detectable partitioning into the carbides.

Incorporating Ru into the matrix increased the melting temperature relative to the base alloy. The highest value, 1335.3 °C, was obtained with 1 wt.% Ru, representing an increase of 18.3 °C over BA. Further additions progressively reduced the melting temperature, although it remained above the BA value of 1317 °C.

Alongside these thermal changes, Ru modified the carbide morphology and mechanical properties. Adding 1 wt.% Ru refined the carbides and improved their spatial distribution, whereas higher additions promoted coarsening, and at 5 wt.%, carbide fragmentation. Across the investigated compositions, carbide microhardness increased from 2309 to 3276 HV_0.01_, while matrix microhardness increased from 266 to 402 HV_0.01_. The combined hardening of these constituents increased the bulk hardness from 37.5 to 43.6 HRC.

These hardness improvements were accompanied by reduced oxidation resistance during air exposure at 1100 °C. Ru additions promoted oxide volatilization and subsurface damage. Mass gain associated with the formation of Cr-, Ti-, and Al-containing oxides occurred concurrently with mass loss through the volatilization of Ru-, Re-, and W-containing oxides. Consequently, net mass changes alone could not reliably describe the oxidation kinetics, making cross-sectional characterization essential.

The cross-sectional observations confirmed that BA and 1Ru retained the highest oxidation resistance, whereas 2.5Ru and 5Ru sustained the most extensive damage. The 2.5Ru alloy developed the thickest retained scale and pronounced cracking, while 5Ru exhibited the greatest internal oxidation area fraction and extensive scale loss. The comparatively thinner retained scale on 5Ru therefore reflected spallation rather than improved protection. These results show that scale thickness must be evaluated alongside scale integrity, internal oxidation, and mass-change behavior.

Under sliding at 1100 °C, all Ru-containing alloys nevertheless exhibited lower wear volumes than BA. The greatest improvement occurred with 1 wt.% Ru, which reduced the wear volume from 1.37 to 0.76 mm^3^ and shifted the dominant wear mechanism from oxidation-induced exfoliation to mild abrasion. Wear volumes increased to 1.13 and 1.20 mm^3^ for 2.5Ru and 5Ru, respectively, as abrasive wear became accompanied by oxidation-induced exfoliation. The 5Ru alloy also showed substantial surface deformation, consistent with load-bearing capacity loss due to subsurface void formation.

The deterioration in wear performance at higher Ru contents highlights the combined importance of carbide morphology and oxidation damage. Coarser TiC particles intensified abrasive wear and hindered the development of protective oxide film, contributing to higher friction and wear. Thus, the greater hardness achieved at higher Ru contents did not compensate for the adverse effects of carbide coarsening and internal oxidation.

The 1Ru alloy therefore provided the most favorable balance of thermal, mechanical, and tribological properties among the investigated compositions. It combined the highest melting temperature of 1335.3 °C with a 35% reduction in carbide size, a more uniform carbide distribution, and increases of 11.8% in HRC hardness relative to BA. At the same time, its matrix and carbide microhardness were 27.4% and 10.4% superior, respectively, compared with BA. These changes were accompanied by a 44.5% reduction in wear volume at 1100 °C and only a moderate deterioration in oxidation resistance. Increasing Ru content beyond 1 wt.% provided additional hardening but intensified carbide coarsening and oxidation damage, limiting the resulting improvement in high-temperature wear resistance.

## Figures and Tables

**Table 1:** Elemental composition of the studied alloys.

Alloy	Elements, wt.%							
	Co	Cr	W	Al	Re	Ru	Ti	C
BA	54.75	20	6	2	6	0	9	2.25
A1Ru	53.75	20	6	2	6	1	9	2.25
A2.5Ru	52.25	20	6	2	6	2.5	9	2.25
A5Ru	49.75	20	6	2	6	5	9	2.25

**Table 2:** Measured TiC peak positions compared with nominal values according to PDF 00-031-1400 card.

Alloy/Reference	Miller Index			
	111	200	220	311
PDF Reference	35.89	41.66	60.41	72.35
BA	36.08	41.92	60.62	72.66
1Ru	36.21	41.96	60.74	72.56
2.5Ru	36.23	41.98	60.74	72.64
5Ru	36.18	41.98	60.69	72.65

**Table 3:** Measured peak positions of cobalt-based solid solution (Co_FCC_) in comparison with nominal ones according to the PDF 01-086-9011 card.

Alloy/Reference	Miller Index		
	111	200	311
PDF Reference	43.95	51.2	91.53
BA	43.92	51.09	91.01
1Ru	43.97	51.087	90.98
2.5Ru	43.89	51.15	91.01
5Ru	43.75	50.98	90.70

**Table 4:** Carbide characteristics in CoCrWReAl-xRu-TiC alloys as a function of Ru content, determined from 39 micrographs per alloy. Figure 4 shows representative micrographs.

№	Alloy	Equivalent Carbide Diameter	Coarsest Carbide Diameter	Carbide Distribution Density	Morphology and Distribution
		μm	μm	mm^−2^	
1	BA	5–9	30–40	1500–1800	High number of fine carbides, with some coarser particles, with some clustering of fine and extra-fine grains. Near-spherical, polygonal and prismatic carbides.
2	1Ru	4–6	20–30	1900–2200	The finest carbides. Very narrow size and distribution in the matrix. Most particles are near-spherical and isometric, with a small number of star-like carbides.
3	2.5Ru	6–10	40–60	1000–1300	Lower number of fine particles, and appearance of coarse carbides. Higher size scattering, particle clustering. Their shape changes to elongated, irregular-shaped, and star-like carbides.
4	5Ru	8–13	50–70	700–1000	The coarsest carbides. Their shape changes to polygonal irregular shape, elongated particles, particle chains, split carbide grains

**Table 5:** Elemental composition of the studied alloys measured during SEM-EDS.

Alloy	Elements, wt.%								
	Co	Cr	W	Al	Re	Ru	Ti	C	Other
BA	51.52	18.55	8.85	1.77	2.2	0	10.53	5.89	0.69
A1Ru	53.73	19.32	7.68	1.82	2.62	0.81	8.61	4.87	0.54
A2.5Ru	52.19	19.24	7.55	1.89	2.29	1.76	8.83	5.63	0.53
A5Ru	48.57	18.66	8.37	1.9	2.18	4.42	10.21	5.17	0.62

## Data Availability

The original contributions presented in this study are included in the article. Further inquiries can be directed to the corresponding authors.
